# Lexico-syntactic interactions during the processing of temporally ambiguous L2 relative clauses: An eye-tracking study with intermediate and advanced Portuguese-English bilinguals

**DOI:** 10.1371/journal.pone.0216779

**Published:** 2019-05-29

**Authors:** Ana Paula Soares, Helena Oliveira, Marisa Ferreira, Montserrat Comesaña, António Filipe Macedo, Pilar Ferré, Carlos Acuña-Fariña, Juan Hernández-Cabrera, Isabel Fraga

**Affiliations:** 1 Human Cognition Lab, CIPsi, School of Psychology, University of Minho, Braga, Portugal; 2 Department of Medicine and Optometry, Linnaeus University, Kalmar, Sweden; 3 Vision Rehabilitation Lab, Centre of Physics and Optometry, University of Minho, Braga, Portugal; 4 Psycholinguistics Research Group, Universitat Rovira i Virgili, Tarragona, Spain; 5 Cognitive Processes & Behaviour Research Group, University of Santiago Compostela, Santiago Compostela, Spain; 6 Department of Psychobiology and Methodology of Behavioral Sciences, University of La Laguna, Tenerife, Spain; University of Ljubljana, SLOVENIA

## Abstract

There is extensive evidence showing that bilinguals activate the lexical and the syntactic representations of both languages in a nonselective way. However, the extent to which the lexical and the syntactic levels of representations interact during second language (L2) sentence processing and how those interactions are modulated by L2 proficiency remain unclear. This paper aimed to directly address these issues by using an online technique (eye-tracking) that is highly sensitive to the lexical and syntactic processes involved in sentence reading. To that purpose, native-speakers of European Portuguese (EP) learning English as L2 at intermediate and advanced levels of proficiency were asked to silently read temporally ambiguous L2 relative clause (RC) sentences disambiguated with a High-Attachment (HA) or Low-Attachment (LA) strategy while their eye-movements were monitored. Since EP and English native speakers differ in the way they process and comprehend this syntactic structure (EP: HA, English: LA), HA preferences were used as a marker of L1 RC syntax interference. Additionally, the cognate status of the complex NP that preceded the RC was manipulated to further analyze how the lexical co-activation of both languages would also affect the syntactic representations of the non-target (L1) language. Results showed cognate facilitation in early and late reading time measures regardless of L2 proficiency, and also that the cognate status of the complex NP impacted L2 reading performance, particularly at lower levels of L2 proficiency. These findings provide compelling evidence for a bilingual reading system that seems to be highly dynamic and interactive not only within each level of processing, but, importantly, across levels of representation. They also suggested that, as the level of L2 proficiency increases, L1 RC syntax interference becomes stronger, in a syntactic parser that seems to operate in a more integrated and nonselective way, with both strategies being equally available to guide L2 reading comprehension. Results are discussed attending to the current models of bilingual syntactic processing.

## Introduction

We live in an increasingly globalized world, where using two or more languages to communicate is becoming the rule rather than the exception [1]. Thus, it is not surprising that a growing number of studies have focused on investigating how two or more languages are acquired, represented, and processed in the bilingual mind. However, despite the fact that acquiring/using a second language (L2) is a very complex process, the majority of studies conducted so far have focused mainly on studying how bilinguals access and/or retrieve individual words from their mental lexicon (e.g., see [2] for a recent review), and not on the way bilinguals combine words with other words to form proper sentences accordingly to the rules of a given grammar to convey meaning.

Although languages do not vary randomly, each has its own set of rules that determines how words can be combined and the relationships that can be established between them, in order to extract the correct meaning. For instance, evidence from the processing of relative clauses (RC) preceded by a complex noun phrase (NP) in sentences like “Someone shot the servant of the actress who was on the balcony” ([3]), have shown that native speakers differ considerably in the way they resolve this syntactic ambiguity across languages (see [4] for a review). Note that, in constructions like this, there are two potential hosts for the RC attachment. It is possible to attach the RC “who was on the balcony” to the first/highest host of the complex NP (i.e., “the servant”), using HA strategy, or to the second/lowest host of the complex NP (i.e., “the actress”), using a LA strategy, thereby leading to different grammatical structures and semantic interpretations. For example, while European Portuguese (EP) native speakers are likely to interpret that the servant was on the balcony, since in this language an HA attachment preference is observed [5], English native speakers would be likely to interpret that it was the actress who was on the balcony, since an LA strategy is preferred in English instead (e.g., [3], [6], [7]). Therefore, studying this grammatical structure offers an excellent opportunity to analyze how the human mechanism responsible for assigning a grammatical structure to a sentence or phrase (parser) works in situations in which individuals master languages whose principles lead to different syntactic and semantic representations, as EP and English. Moreover, since previous works have shown that words that share meaning and form (orthography and/or phonology) across languages (i.e., cognate words, such as *actress* and *actriz* in English and EP, respectively) are processed faster and more accurately than noncognate words (i.e., words that share meaning, but not form across languages, such as *servant* and *criada* in English and EP, respectively; see [8] and [9] for reviews), embedding these words in the complex NP of the RC structures can also provide an excellent opportunity to further explore how the lexical and syntactic levels of representation interact in the bilingual mind during L2 sentence processing, a largely unexplored issue in the bilingual and L2 acquisition literature.

The lack of research in this area is quite surprising since current models of sentence processing in monolinguals assume that multiple sources of information (e.g., lexical, syntactic, semantic) should be used and appropriately integrated to achieve a correct interpretation of what is meant (e.g., see [6], [10], [11] for different proposals about the mechanisms and the stage at which each kind of information is accessed and processed). However, on the L2 sentence processing literature, researchers have focused mainly on exploring whether the facilitation effect observed for cognates presented in isolation persists when they were presented in sentence contexts (i.e., on the lexical level of sentence processing, see [12] for a review), or on exploring to what extent the syntactic representations of both languages are activated when a bilingual is reading in their L2 (i.e., on the syntactic level of sentence processing, see [13] for a review), hence neglecting the interactions that can be established between the lexical and the syntactic levels of representation during L2 sentence processing.

Research on the lexical level of L2 sentence processing, conducted mainly through the use of cognate words embedded in sentence contexts (e.g., [12], [14], [15], [16], [17], [18], [19], [20]), provide strong support for a nonselective view of lexical access in bilinguals, i.e. for the idea that both languages are activated in the bilingual mind even in situations that provide readers with much more information about the language in use, which could restrict strongly the level of lexical cross-language activation (see for example [9] for a model assuming a nonselective or integrated view of lexical processing in bilinguals). Evidence for a nonselective view of syntax in bilinguals has been also observed from two other lines of studies. One line, known as *language transfer*, focused on exploring how the syntactic properties of the L1 affect the syntactic processing of the L2 (e.g., [4], [7], [21], [22], [23], [24], [25], [26]), and the other, known as *syntactic priming*, on analyzing the extent to which the production of a sentence with a particular syntactic structure in one language (typically the L1) affects the production of another sentence with the same syntactic structure in the other language (typically the L2), by using a syntactic priming paradigm (e.g., [27], [28]).

Specifically, the first kind of studies, have shown that bilinguals often use information from the L1 to construct and to process the syntactic structures of the L2, hence suggesting that L2 parsing is affected by the properties of the L1 grammar. For instance, in a seminal eye-tracking study, Frenck-Mestre [24] showed that, while native speakers of Spanish, who were non-proficient learners of French, showed an HA preference in reading L2 (French) temporally ambiguous RC sentences (the RC preference of both languages), native speakers of English, who were also non-proficient learners of French, showed a trend towards an LA preference (the RC preference of English) in reading the same sentences. Frenck-Mestre [24] interpreted these findings as a marker of the influence of L1 syntactic characteristics on L2 sentence processing, although subsequent studies with highly proficient L2 learners (e.g., [23], [25], [26]) failed to show this effect. These findings have been also used to suggest that the impact of the L1 over the L2 seems to decline as proficiency increases, which is in accordance with the Competition Model (CM) of MacWhinney ([29], [30]), claiming that, at the first stages of L2 acquisition, learners transfer the grammatical features of the L1 to help them comprehend and produce L2 structures. However, as the level of proficiency increases, the CM states that effects of the L1 over the L2 tend to diminish and L2 learners would become more sensitive to the syntactic specificities of the L2. Hence, accordingly to the CM model, the acquisition of syntax in a L2 is characterized by a progressive movement going from a maximal syntax share (i.e., L1 determines L2 syntactic processing) at lower levels of L2 proficiency to a minimal syntax share (i.e., L2 specificities are recognized and L1 characteristics do not interfere with L2 syntactic processing) at higher levels of L2 proficiency.

Research from the second line of studies has also provided strong evidence for a nonselective or shared view of syntax in bilinguals (e.g., [13], [28], [31], [32], [33], [34], [35], [36]). For example, in one of the first syntactic priming studies with bilinguals, Hartsuiker et al. ([13]) used a dialogue game to explore L1-L2 syntactic interactions in the production of grammatical structures that were shared and not shared between languages. Specifically, in that task, the authors had a confederate producing a Spanish (L1) sentence with a particular structure (passive, active, intransitive) to describe a picture, and immediately after they asked Spanish (L1)–English (L2) bilinguals to describe another picture in English (L2). Results showed that reliable syntactic priming effects were only observed for passives as participants produced significantly more L2 passives after hearing an L1 passive sentence than after hearing an L1 active or intransitive sentence. Because the passive structure exists in both languages, Hartsuiker et al. concluded that, as long as a given grammatical structure is similar enough across languages, their syntactic representations are shared between languages. Note that other studies have also shown that syntactic priming effects were syntactic in nature, as they were still observed when none of the items used in the prime and target sentences were repeated, and even in the absence of any thematic similarity between them (e.g., [37], [38], [39]).

Nonetheless, recent studies have shown that syntactic priming effects can be boosted if translation equivalents were used in the sentences (e.g., *geven*[give] in the Dutch prime sentence and ‘give’ in the English target sentence vs. *verkopen*[buy] in the Dutch prime sentence and ‘give’ in the English target sentence), particularly when participants were asked to produce the target sentences in their L2 (e.g., [31], [32], [35], [36]). This boost to syntactic priming was explained based on the co-activation of both syntactic and conceptual representations for grammatical structures that are not only shared across languages but that also contained translation equivalents relative to non-translation equivalents (unrelated words) (see [35], [37] for details). Note, however, that although in these studies some of the translation equivalents used also share form across-languages (i.e., they are cognates, such as *geven*-give in the above mentioned example), the lexical (word-strata) level of processing was largely ignored in the explanation of the results, notwithstanding that Cai et al. [32] have recently demonstrated that the syntactic boost for cognates did not arise from the lemma level of processing.

Another variable that seems to modulate the magnitude of the syntactic priming effect is L2 proficiency (e.g., [32], [34], [40]). For instance, Bernolet et al. [40], in a recent study with Dutch (L1)–English (L2) late bilinguals with different levels of L2 proficiency, using head-nouns with the same meanings (e.g., *ei*[egg]–egg) or with different meanings (e.g., *paard*[horse]–egg) in a dialogue game similar to the one used by Hartsuiker et al. [13], showed stronger syntactic priming effects for more proficient compared to less proficient bilinguals in both conditions. They also showed that low-proficiency bilinguals benefited more from the use of translation equivalents than high-proficiency bilinguals. These results led the authors to conclude that, at lower levels of L2 proficiency, bilinguals represent the syntactic structures of both languages separately (i.e., that, at early stages, syntactic representations are more ‘specific’ and more dependent on the lexical properties of the items used), and only when they become more proficient is there a shift from ‘item specific’ syntactic representations to abstract and shared syntactic representations. These results are interesting, although, contrary to the CM ([29], [30]), they suggest that, as the level of L2 proficiency increases, the influence of the L1 syntax over L2 sentence processing becomes stronger and not weaker. Moreover, since the findings of this and other studies (e.g., [32], [39], [35]) have also shown that the magnitude of syntactic priming is modulated by the lexical properties of the items used in the sentences, it is reasonable to assume that the lexical and the syntactic levels of representation interact during L2 sentence processing in a bilingual reading system that is not only interactive within each level of processing, as the vast amount of lexical and syntactic L2 sentence processing studies demonstrate, but, importantly, across levels of representation (i.e., lexical and syntactic). However, studies aimed to analyze how the lexical and the syntactic levels of representation interact during L2 sentence processing, and how these interactions were modulated by L2 proficiency, are scarce.

To the best of our knowledge, only two recently published studies have directly addressed these issues by manipulating the type of translation equivalents used in the sentences (cognates vs. noncognates) and the level of L2 proficiency ([41], [42])—note that in the Cai et al. [32] study, cited above, the authors used cognates vs. unrelated words (e.g., gave vs. donated, respectively) embedded in the prime sentences (e.g., /The cowboy gave/donated the sailor a book/) in a group of highly Cantonese (L1)-Mandarin (L2) bilinguals. In Hopp’s [41] study, the author asked German (L1)-English (L2) late bilinguals with intermediate-to-high levels of L2 proficiency to read English sentences containing either reduced RCs whose surface word order corresponds to the regular SOV order in German (e.g., When the doctor Sarah ignored tried to leave the room the nurse came in all of a sudden) and non-reduced (main clauses) RCs that do not overlap with the canonical German surface word order (e.g., The doctor who Sarah ignored tried to leave the room when the nurse came in all of a sudden), and two other sentences with overt relative pronouns as controls (see [41] for details). Critically, the first verb in the sentences could be either a cognate (e.g., ignored) or a noncognate (e.g., visited) which allowed the author to examine whether the cross-language activation of the L1 grammatical structure was affected by the cross-language lexical activation generated by the use of cognates relative to noncognates in the sentences. Two eye-tracking studies were reported in a sentence reading task presented exclusively on the L2 (Experiment 1) and in a mixed-language context (Experiment 2). In both experiments, a cognate facilitation effect was found in early reading time measures regardless of L2 proficiency. Results from Experiment 1 also showed that only low-proficiency bilinguals activated the L1 word order in first-pass reading measures. In Experiment 2, all bilinguals showed L1 syntactic co-activation, though only for sentences containing noncognates. Hopp ([41]) interpreted these findings in accordance with capacity models of L2 reading comprehension (e.g., [43], [44], [45]), claiming that the activation of the L1 syntax would become more noticeable in conditions of increased lexical processing demands (i.e., for noncognates), since the stronger cross-language lexical activation observed for cognates eases processing and frees cognitive resources for inhibiting L1 syntax more effectively.

In the same vein, Soares et al. ([42]) embedded cognates and noncognates in the complex NPs of ambiguous RC sentences to analyze how the cognate status of the complex NP affected the RC syntactic resolutions of EP (L1)-English (L2) intermediate and advanced bilinguals who were asked to perform an L2 sentence completion task. Since EP and English native speakers differ in the way they resolve this syntactic ambiguity (EP: HA vs. English: LA), the number of HA completions was used as a marker of L1 syntax interference (see [42] for details). The cognate status of the complex NP was manipulated in four experimental conditions, in such a way that both nouns in the complex NP could correspond to cognates (Cognate-Cognate condition, C-C), the first to a cognate and the second to a noncognate (Cognate-NonCognate condition, C-NC), the first to a noncognate and the second to a cognate (NonCognate-Cognate condition, NC-C), or both to noncognates (NonCognate-NonCognate condition, NC-NC). The authors found that participants showed a global preference to use the LA strategy to resolve the L2 RC ambiguities (i.e., they produced more LA than HA RCs to complete the sentences), regardless of their level of L2 proficiency. Moreover, the analysis of the HA responses (indicative of L1 syntax interference) across the cognate conditions also showed that both groups revealed stronger L1 RC interference (i.e., more HA completions) in the NC-NC condition than the C-C condition, and that the NC-C condition induced stronger L1 RC interference than the C-NC condition. Two explanations were proposed to interpret these results: (i) a *cognitive load hypothesis*, arguing that, since cognates could have generated stronger lexical co-activation of the non-target language (L1), this lexical co-activation could have spread to syntactic co-activation which in turn could have yield higher levels of cross-syntactic competition for RC attachment (bear in mind that English and EP show different RC attachment preferences), which might have contributed to overload processing, hence stimulating the use of a local (LA) over a non-local (HA) strategy; and (ii) a *resource capacity hypothesis*, claiming, in line with Hopp’s proposal ([41]), that because noncognates are harder to process than cognates (as they do not benefit from form activation in both languages), this might have not released the resources necessary to inhibit L1 syntax effectively, thus making L1 RC syntax interference to be more noticeable for noncognates than cognate conditions. However, since Soares et al. ([42]) used an offline task, it was not possible to disentangle these two alternative hypotheses in the explanation of the results.

In the present paper we aimed to further explore the same research questions by using an well-known on-line technique (eye-tracking) that provides a continuous recording of sentence processing (e.g., [46], [47]). Note that if the cognitive load hypothesis explains the fact that more LA completions were observed in the C-C than in the NC-NC conditions, then longer reading times would be expected in the C-C than in the NC-NC condition, and, importantly, differences between HA and LA readings would be only noticeable in the NC-NC and not the C-C condition. Indeed, if cognates induce stronger cross-syntactic competition for RC attachment as the a cognitive load hypothesis states, this would mean that both strategies would be equally available for reading, thus making that the differences between HA and LA reading times not reach statistical significance. Conversely, if cognates ease processing and release cognitive resources for L1 RC inhibition, as the resource capacity hypothesis predicts, longer reading times would be observed in the NC-NC than the C-C condition, and, importantly, the differences between HA and LA readings would be only observed in the C-C and not in the NC-NC condition. Thus, both hypotheses predict quite different results regarding what would be expected in the NC-NC and in the C-C conditions.

Moreover, it is also worth noting that, while differences between the C-NC and the NC-C conditions (i.e., a cognate position effect) are not expected by the resource capacity hypothesis, as both conditions represent similar lexical demands (note that each of them entails one cognate and one noncognate), a cognate position effect would be expected according to the cognitive load hypothesis, with the C-NC condition producing not only longer reading times than the NC-C condition, but also stronger L1 RC interference. Indeed, as advanced by Soares et al. ([42]), it is possible that, when the cognate is located at the L1 RC preferential position (first), it would activate the L1 RC preference more strongly than when it is located at the non-preferential position (second), thus justifying not only longer times in the C-NC than in the NC-C conditions, but also that the HA-LA differences only reached statistical significance in the NC-C condition.

Finally, as in Soares et al. study ([42]), in this paper we also intend to examine how L2 proficiency affects lexico-syntactic interactions during L2 RC sentence processing by testing two groups of native speakers of EP learning English as L2 (intermediate vs. advanced). High-proficiency learners are expected not only to present faster reading times than low-proficiency learners, but, importantly, to demonstrate a more L2 native-like way of processing, as predicted by the CM ([29], [30]). This does not mean, however, that L1 syntax co-activation will not be noticed at higher levels of proficiency as recent studies demonstrated (e.g., [13]). Nevertheless, in line with the predictions of the CM ([29], [30]) and also with the findings of Hopp ([41]), we expect to observe stronger L1 RC syntax interference for intermediates than for advanced L2 learners.

## Ethics statement

The experiments presented here have been carried out in accordance with the code of ethics of the World Medical Association (Declaration of Helsinki) and were approved by the Ethics Committee for Human Research of University of Minho (reference number 035–2014) (Braga, Portugal) and also by the Ethics Committee of the Department of Psychology, Royal Holloway University of London (reference number 2015/005). Written consent was obtained from all participants.

## Method

### Participants

Thirty intermediate (22 females, *M*_age_ = 20.3 years, *SD* = 8.57) and 30 advanced (26 females, *M*_age_ = 22.5, *SD* = 6.00) learners of English as L2, recruited from English teaching institutions in Portugal, took part in the experiment. All participants had normal or corrected-to-normal vision and were native-speakers of EP. The levels of L2 proficiency were obtained from the English learning institutions in which participants were enrolled. Intermediate learners presented a B_1_ level of proficiency while advanced learners presented a C_1_ level of proficiency according to the Common Reference Levels of Language Proficiency (CRLLP) of the Council of Europe (see http://www.coe.int/t/dg4/linguistic/cadre1_en.asp). The use of intermediate rather than lower-level learners in this study was to ensure that L2 learners would be sensitive to the grammatical structure under study. Furthermore, since in this work we also aimed to analyze at what extent the results recently observed by Soares et al. (42) with an offline sentence completion task were also observed in the on-line sentence reading task used in the present paper, we also keen to ensure that the L2 proficiency groups used in both experiments were highly equivalent.

Information about participants’ language background was obtained using the on-line version of the Language History Questionnaire (LHQ, [48]). Responses to this questionnaire revealed that all the intermediate and advanced learners were firstly exposed to English before the age of 10 (*M*_intermediate_ = 8.7 years, *SD* = 2.93; *M*_advanced_ = 8.7 years, *SD* = 2.04, *t*(57) = .12, *p* = .90). Also, intermediate learners reported spending fewer hours per day using their L2 in activities such as watching TV, listening to music, or using internet than advanced learners (*M*_intermediate_ = 3.7, *SD* = 2.44; *M*_advanced_ = 7.6, *SD* = 7.17, *t*(41) = -2.41, *p* = .021), and also a lower number of years of English training (*M*_*intermediate*_ = 7.5 years; *SD* = 2.15; *M*_advanced_ = 10.7 years; *SD* = 4.16, *t*(56) = -3.64, *p* < .001). The subjective ratings of L2 reading, writing, speaking, and listening skills obtained for each group from the LHQ are presented in Table 1.

**Table 1:** Means and Standard Deviations (in brackets) for the subjective ratings of reading, writing, speaking, and listening skills in the two groups of L2 learners.

L2 groups	Reading	Writing	Speaking	Listening
Intermediate	5.52 (0.85)	4.89 (1.16)	5.04 (1.29)	5.44 (1.09)
Advanced	6.10 (0.71)	5.37 (0.93)	5.60 (0.97)	6.03 (0.89)

7-point Likert scale ranging from 1 “very poor” to 7 “native-like”.

As expected, advanced learners reported higher self-ratings of reading, *t*(55) = -2.81, *p* < .001; writing, *t*(55) = -1.73, *p* = .09; speaking, *t*(55) = -1.88, *p* = .07; and listening L2 skills, *t*(55) = -2.25, *p* < .05, although the differences in writing and speaking abilities were only marginally significant. Consistent with this, the results from the LexTALE task ([49]), also revealed that advanced learners were better at recognizing English words than intermediate learners (*M*_intermediate_ = 64.8%, *SD* = .08; *M*_advanced_ = 71.6%, *SD* = .10, *t*(55) = -2.97, *p* < .001). Moreover, it is also important to stress that, since previous research showed that L2 sentence processing is cognitively more demanding than L1 sentence processing, and also more dependent on the cognitive resources available, namely working memory (e.g., [43], [50], [51]), we additionally assessed participants’ working memory capacity to assure that it was equivalent across groups, and did not affect the results inadvertently. To that purpose, we used a Digit Span Task performed both in EP and English, as Arêas da Luz Fontes and Schwartz ([52]). In this task participants heard a sequence of numbers in a series that increased from three to eight digits and, after each sequence, they were asked to recall the numbers heard in the correct order by typing them in the space provided on the computer screen. The only difference between the EP and English versions of the task was that in the EP version the numbers were presented in the native language for each group. In each version, the task involved 12 trials, two for each digit length, and no time limit was imposed to recall each sequence, although participants were stimulated to respond as quickly and accurately as possible. The results showed no differences between L2 groups on the working memory capacity in either EP (*M*_intermediate_ = 7.0, *SD* = 1.56; *M*_advanced_ = 7.3, *SD* = 1.70, *t*(58) = -.59, *p* = .56), or the English (*M*_intermediate_ = 6.6 years, *SD* = 1.32; *M*_advanced_ = 7.1 years, *SD* = 1.98, *t*(58) = -1.11, *p* = .27) versions of the task.

Finally, a group of 30 native-speakers of English (27 females, *M*_age_ = 20.7 years; *SD* = 2.59), with no prior knowledge of the EP language, were also recruited to participate in the study as a control. They were recruited from the Royal Holloway University of London (London, England) and took part in the experiment in exchange of financial compensation. All of them had normal or corrected-to-normal vision.

### Materials

The experimental stimuli consisted of a set of 96 L2 RC temporally ambiguous sentences based on the materials used by Soares et al. ([42]). In each sentence, the subject of the matrix verb was a proper noun or personal pronoun, and the direct object consisted of a complex NP containing two nouns (N1 and N2) connected by the genitive marker “of”, followed by a relative clause (RC). In the complex NP both nouns were animate or inanimate to control for animacy effects on RC attachment (see [5] or [53] for studies showing how the animacy of the constituents affects RC attachment). The RC, introduced by the relative pronoun who/that, was forced to attach either to the first (N1) or to the second noun (N2) of the complex NP by a critical word (N3) that was semantically related to either N1 or N2 using an HA or LA strategy, respectively. For instance the sentence “Britney recognized the guard(N1) of the prisoner(N2) who had been honoured(N3) for his braveness” was disambiguated with an HA strategy since the semantic context in the RC is more likely associated with the first- (N1) than the second-host (N2) in the complex NP, while the sentence “Britney recognized the guard(N1) of the prisoner(N2) who had been sentenced(N3) to death penalty” was disambiguated with an LA strategy since the semantic context in the RC is more likely associated with the second- (N1) than the first-host (N2) in the complex NP that precedes the RC. Therefore, half of the sentences presented an HA strategy of disambiguation (48 sentences), while the other half presented an LA strategy. It is worth noting here that, although previous studies on L2 RC attachment adopted a number agreement strategy in the disambiguation of the sentences (e.g., [4], [23], [25]), in our study we opted for a semantic/pragmatic disambiguation strategy, as in Soares et al. ([42]), because the use of the number strategy disrupts the typical RC attachment observed in EP as shown by a previous sentence completion EP study (see [54]).

The 48 HA and the 48 LA sentences were additionally assigned to four experimental conditions according to the cognate status of the nouns embedded in the complex NP. Thus, in each group, 12 sentences presented a cognate word both in the N1 and N2 (C-C condition), 12 presented a cognate in the N1 and a noncognate in the N2 (C-NC condition), 12 presented a noncognate in the N1 and a cognate in the N2 (NC-C condition), and, finally, 12 presented a noncognate both in the N1 and N2 (NC-NC condition). Importantly, the sentences disambiguated with an HA and an LA strategy in each cognate condition were exactly the same except in the RC that contained the critical word and the semantic context that disambiguated the sentence in each of its potential directions (i.e., with an HA or LA strategy). For instance, the abovementioned sentence disambiguated either with an HA or an LA strategy was from the C-C condition since both nouns in the complex NP are English-EP cognates (*guard* and *guarda*, and *prisoner* and *prisioneiro* in English and EP, respectively). The same applied in all the other conditions. Furthermore, the C and NC words embedded in the complex NPs (N1 and N2) and the critical word in the RC (N3) that forced the disambiguation towards the first- (N3_HA_) or the second-noun of the complex NP (N3_LA_) were matched within and across-conditions considering grammatical category (PoS) (all nouns), length (number of letters), lexical frequency (Zipf scale measure (see [55]), and level of orthographic overlap as indexed by the Normalized Levenshtein Distance (NLD) measure as provided by the NIM software ([56]). In addition, this control was established not only considering the lexical properties of the English words used in the experimental sentences, but also the lexical properties of its translation equivalents into EP for an additional cross-language control. Data on the PoS, lexical frequency, and length of the words were taken from the N-Watch ([57]) and the SUBTLEX-UK ([55]) databases for English, and from the Procura-PALavras (P-PAL; [58]) and the SUBTLEX-PT ([59]) databases for EP. Table 2 shows the psycholinguistic characteristics of the words (N1, N2, N3_HA_ and N3_LA_) used in the experimental sentences both in English and in EP.

**Table 2:** Means and Standard Deviations (in brackets) of the psycholinguistic characteristics of N1, N2, and N3 words used in the experimental sentence in the four cognate conditions.

Sentence condition	Psycholinguistic characteristics	N1	N2	N3_HA_	N3_LA_
**C-C**	**Length English**	7.1 (1.7)	6.4 (1.2)	6.1 (2.3)	6.7 (1.4)
	**Length EP**	7.5 (1.8)	6.8 (2.0)	7.8 (2.2)	8.3 (2.0)
	**Frequency English**	3.9 (0.9)	4.1 (0.4)	4.1 (0.6)	4.3 (0.6)
	**Frequency EP**	4.1 (0.7)	4.1 (0.6)	3.8 (0.7)	3.7 (1.1)
	**Orthographic overlap**	0.6 (0.2)	0.7 (0.2)	0.2 (0.1)	0.2 (0.1)
**C-NC**	**Length English**	6.4 (3.3)	6.2 (1.8)	5.8 (1.7)	5.8 (1.6)
	**Length EP**	6.9 (3.3)	8.0 (2.2)	7.6 (2.5)	7.3 (2.3)
	**Frequency English**	4.4 (0.7)	4.3 (0.5)	4.1 (1.1)	4.2 (0.7)
	**Frequency EP**	4.3 (0.7)	4.4 (0.8)	3.2 (1.0)	3.8 (0.9)
	**Orthographic overlap**	0.6 (0.1)	0.2 (0.1)	0.2 (0.1)	0.2 (0.1)
**NC-C**	**Length English**	6.1 (1.2)	7.3 (1.7)	5.3 (1.1)	6.8 (2.1)
	**Length EP**	5.9 (1.7)	7.4 (2.4)	6.8 (2.5)	7.6 (2.1)
	**Frequency English**	4.2 (0.5)	4.4 (0.5)	4.6 (0.7)	4.0 (0.8)
	**Frequency EP**	4.3 (0.5)	4.4 (0.3)	4.2 (0.7)	3.8 (0.7)
	**Orthographic overlap**	0.2 (0.1)	0.7 (0.1)	0.1 (0.1)	0.2 (0.1)
**NC-NC**	**Length English**	5.0 (2.1)	5.6 (1.8)	6.4 (1.6)	5.8 (1.8)
	**Length EP**	5.6 (2.0)	5.9 (1.6)	6.2 (1.5)	6.4 (1.2)
	**Frequency English**	4.3 (0.6)	4.6 (0.5)	3.8 (0.85)	4.2 (0.71)
	**Frequency EP**	4.1 (1.2)	4.5 (0.5)	3.8 (1.1)	4.1 (1.0)
	**Orthographic overlap**	0.1 (0.1)	.09 (0.1)	0.2 (0.1)	0.2 (0.2)

C-C, Cognate-Cognate; NC-C, NonCognate-Cognate; C-NC, Cognate-NonCognate; NC-NC, NonCognate-NonCognate; N1, first-noun of the complex noun phrase; N2, second-noun of the complex noun phrase; N3_HA_, critical word that disambiguates the sentence with a high attachment strategy; N3_LA_, critical word that disambiguates the sentence with a low attachment strategy; EP, European Portuguese.

Several analyses were performed on the materials to assure that the words used in the sentences were well controlled, both within and between languages, and across experimental conditions. Specifically, the paired samples *t*-tests performed for the N1 and the N2 comparisons in each experimental condition and language revealed that the nouns embedded in the complex NPs did not differ either in number of letters (all *p*s > .13) or word frequency (all *p*s > .11) in English or EP. As expected, the only statistically significant difference was on the level of orthographic overlap, as the N1 and the N2 were significantly different in the C-NC and NC-C conditions (*p*s < .001), but not in the C-C and NC-NC conditions (*p*s > .44). Additionally, the ANOVAS conducted for the N1, as well as for the N2 across cognate conditions, revealed the absence of any statistical differences in all the psycholinguistic variables under analysis, except for the level of orthographic overlap for both the N1, *F*(3, 44) = 48.537, *p* < .001 and the N2, *F*(3, 44) = 79.786, *p* < .001. As expected, these effects showed that, while the N1 words differ statistically on the comparisons between the C-C and the NC-C conditions, and the C-C and NC-NC conditions (all *p*s < .001), they did not differ in the comparisons between the C-C and C-NC conditions, or in the NC-C and NC-NC conditions (all *p*_s_ = 1.00). Similarly, the N2 words did not differ significantly in the comparisons between the C-C and NC-C, or the C-NC and NC-NC conditions (all *p*_s_ = 1.00), but they did differ in the C-C and the C-NC, as well as in the C-C the NC-NC comparisons (all *p*s < .001). Besides, the analyses conducted on the lexical properties of the N3 words used to disambiguate the sentences with an HA and an LA strategy in each language revealed no significant differences when considering the PoS (all *p*s > .47), the frequency of occurrence of the words (all *p*s > .15), or the number of letters (all *p*s > .31), though a marginal significant difference in the number of letters was observed in the NC-C condition, showing that the English words used in the HA sentences tended to be slightly longer that the ones used in the LA sentences (*p* = .06). Additionally, the ANOVAs conducted both for the N3_HA_ and the N3_LA_ nouns across cognate conditions showed that the N3_HA_ and the N3_LA_ nouns did not differ significantly either in length (all *p*s > .31) or word frequency (all *p*s > .31) in each language, nor in the level of orthographic overlap (note that the N3 words used in the HA and LA sentences presented a level of orthographic overlap similar to those presented by the noncognates used both in N1 and N2, see Table 2).

Finally, it is also important to mention that the experimental sentences used were selected from a preliminary study conducted in both English and EP to ensure that the plausibility of the HA and LA sentences was equivalent within and across conditions in each language. Thirty native-speakers of English (23 females, *M*_age_ = 23.3 years, *SD* = 9.93) and 30 native-speakers of EP (26 females, *M*_age_ = 25.6 years, *SD* = 9.11) participated in the English and EP plausibility study, respectively. They were recruited from the same population of participants (i.e., university students) as those of the eye-movements experiments reported here, though none of them participated in both studies. Following Fernández ([4]) procedures, two versions of the same sentence were constructed in each language. These two versions were constructed by associating the RC to each of the nouns of the complex NP (N1 and N2), thereby creating a semantically congruent and a semantically incongruent version of the same sentence. For instance, the plausibility of the sentence “Britney recognized the guard of the prisoner who had been honoured for his braveness” was assessed by creating the sentences “The guard had been honoured for his braveness” (congruent) and “The prisoner had been honored for his braveness” (incongruent) that were presented in pairs to the participants. For each pair, participants were asked to rate each sentence for plausibility on a 5-point scale ranging from 1 (“not plausible”) to 5 (“very plausible”). They were also instructed to base their judgments on real-world plausibility and that in each pair the plausibility of one sentence was not necessarily different from the plausibility of the other. From the plausibility studies conducted, we selected the HA and LA sentences that presented high plausibility rates (> 80%) in both languages. Table 3 presents the subjective ratings of plausibility obtained for the 96 experimental sentences used in the eye-tracking experiments reported in this paper both in the English and EP version of the sentences.

**Table 3:** Means and Standard Deviations (in brackets) of the plausibility ratings obtained for the experimental sentences in English and EP by cognate condition.

Language	Cognate Condition	HA	LA
**English**	**C-C**	4.6 (0.3)	4.7 (0.3)
	**C-NC**	4.7 (0.3)	4.7 (0.2)
	**NC-C**	4.6 (0.2)	4.6 (0.4)
	**NC-NC**	4.5 (0.5)	4.7 (0.2)
**EP**	**C-C**	4.8 (0.1)	4.8 (0.2)
	**C-NC**	4.8 (0.2)	4.8 (0.1)
	**NC-C**	4.8 (0.2)	4.7 (0.3)
	**NC-NC**	4.8 (0.2)	4.9 (0.1)

5-point Likert scale ranging from 1 “not plausible” to 5 “very plausible”; C-C, Cognate-Cognate; NC-C, NonCognate-Cognate; C-NC, Cognate-NonCognate; NC-NC, NonCognate-NonCognate.

The *t*-tests for paired samples conducted in each language confirmed that the HA and LA sentences were equally plausible in each cognate condition (*p*s > .22). Moreover, the ANOVAs conducted to compare the HA and the LA ratings across cognate conditions in each language showed that the plausibility of the HA and LA sentences did not differ significantly in either English (*p*s > .82) or EP (*p*s > .23). Finally, in addition to the 96 experimental sentences, we also constructed 96 sentence fillers to distract participants from the grammatical structure under study, and 192 comprehension questions (e.g., “Who had been honoured?”) with two options (e.g., “the guard/ the prisoner”) to ensure that participants were reading for comprehension. Four lists of materials were created by counterbalancing the experimental sentences in each cognate condition according to the disambiguation strategy used (participants were only presented with one version [HA or LA] of the sentence) and to the presentation or not of a comprehension question following a given experimental sentence. Ten practice items were also included to familiarize participants with the task.

### Procedure

The 144 sentences (48 experimental plus 96 fillers) were presented in L2 (English) in a silent reading task with eye-movements monitoring. Participants were seated at a distance of approximately 60 cm from the computer screen. They were asked to read each sentence silently at their normal reading pace, making sure to comprehend the sentence since they would have to occasionally respond to a comprehension question (in a third of the cases, 48 sentences in total, half experimental and half fillers). Sentences were presented in a single line of text, aligned to the left side at the center of a 22'' inch LCD monitor (Dell P2210) in a black 24-point Courier New font. At the beginning of each trial, a fixation cross was presented on the left side of the screen indicating the position where the first word of the sentence was going to appear. After reading each sentence, participants had to press a key to proceed to the next sentence. If a comprehension question appeared, they were required to decide which of the options presented was correct by using the computer’s mouse, before proceeding to the next trial. There was no time limit to read the sentences or to answer to the comprehension questions, although participants were encouraged to respond as quickly and accurately as possible. Sentences were presented in a pseudo-random order to guarantee that each experimental sentence was interspersed by two fillers. Eye movements were monitored by using a binocular eye-tracker running at 250Hz (RED250, SMIGmb Germany), spatial resolution < 0.4°. Calibration was done using a 9-point RED method. Data from the two L2 learner groups were collected individually in the Human Cognition Laboratory (School of Psychology, University of Minho, Portugal), whereas data from the control group were collected in the Wolfson Laboratory (Department of Psychology, Royal Holloway University of London, England). The procedure took about 45 min in the L2 learner groups and 30 min in the control group.

## Results

Data were analyzed during first-pass reading in two regions of interest in the sentences: the complex NP that preceded the RC in which the cognates and noncognates words were embedded (the N1+N2 region), and the N3 region in the RC that corresponded to the first content word that disambiguated the sentence with an HA or an LA strategy. For each region, five eye-movement measures were computed, since these are the most commonly analyzed in sentence processing (e.g., [14], [16], [17], [20]) and are thought to index different stages of processing ([46], [47]): *First Fixation Duration* (FFD), the length of time the eyes fixate on the target region the first time they land on it, which is typically associated with processes of lexical access; *First-Pass Reading Times* (FPRT), the sum of all fixations during first-pass reading on the target region, indexing syntactic processing; *Total-Reading Times* (TRT), the sum of all fixations across all re-readings on the target region, indexing processes of language integration; *Regressions Out* (RO), the proportion of regressive eye-movements out of the target region during first-pass reading, indexing language reanalysis; and *Skipping Rates* (SR), the proportion of skipping on the target regions.

The FFD, FPRT, and TRT measures were divided by the number of characters in the region, to obtain the number of milliseconds (ms) per character. Although the length of the N1, N2, and N3 words in the sentences was equivalent across conditions (though on the NC-C condition the N3 words used in the HA sentences tended to be slightly longer than the N3 words used in the LA sentences; see the materials section), we decided to adopt this procedure to reduce error variance by making different items roughly comparable with each other within and across sentence regions (bear in mind that the N1+N2 region is larger than the N3 region; see [7] for a similar procedure). It is also worth noting that, prior to the analyses, trials were removed if the fixation duration on the target region was less than 80 ms, a common practice in reading eye-tracking studies (e.g., [46], [47]). FPRT were excluded from the analyses whenever that region was skipped on the first-pass reading. Trials that deviated 2.5 *SD*s below or above the mean of the trials in each experimental condition were also eliminated. Altogether, this resulted in the removal of 3.2% of the data in the control group, 3.5% of the data in the intermediate L2 group, and 3.9% of the data in the advanced L2 group. Table 4 presents the per character reading times of the FFD, FPRT, and TRT measures, as well as the proportion of RO during first-pass reading, for both the N1+N2 and the N3 sentence regions. Skipping rates are not presented because they were very few and equally distributed across conditions in each group. Specifically, in the N1+N2 region SR ranged from .02 to .04 in the intermediate group (*p*s > .45), from .01 to .02 in the advanced group (*p*s = .84), and from .02 to .04 in the control group (*p*s > .80). In the N3 region, SR ranged from .04 to .12 in the intermediate group (*p*s = .39), from .04 to .08 in the advanced group (*p*s = .84), and from .09 to .17 in the control group (*p*s = .74).

**Table 4:** Means and Standard Deviations (in brackets) of the FFD, FPRT, and TRT measures, and of the proportions of RO in the complex NP region (N1+N2) and in the critical word that disambiguates the sentence (N3 region) by experimental condition and participant group.

Sentence region		N1+N2								N3							
Cognate conditions	Measures	FFD		FPRT		TRT		RO		FFD		FPRT		TRT		RO	
	Disambiguation Groups	HA	LA	HA	LA	HA	LA	HA	LA	HA	LA	HA	LA	HA	LA	HA	LA
**C-C**	Intermediate	15.2 (4.3)	16.2 (5.2)	52.5 (13.9)	51.8 (17.5)	99.8 (51.3)	96.2 (33.8)	0.4 (0.3)	0.4 (0.3)	55.4 (13.1)	45.2 (12.4)	66.2 (20.9)	57.9 (10.1)	108.3 (53.0)	91.6 (42.7)	0.2 (0.2)	0.2 (0.2)
	Advanced	15.0 (2.9)	13.8 (3.1)	51.9 (11.1)	47.2 (13.3)	90.3 (23.5)	94.8 (43.5)	0.4 (0.2)	0.4 (0.3)	47.6 (10.9)	38.0 (7.2)	62.7 (29.7)	45.3 (11.7)	104.0 (34.1)	70.8 (23.2)	0.2 (0.2)	0.2 (0.2)
	Control	11.6 (2.3)	11.7 (2.3)	35.4 (8.8)	32.9 (8.7)	59.5 (21.0)	63.0 (22.7)	0.3 (0.2)	0.3 (0.3)	35.2 (7.5)	31.0 (5.2)	38.4 (12.4)	36.3 (6.3)	61.9 (19.7)	54.9 (18.9)	0.2 (0.3)	0.3 (0.2)
**C-NC**	Intermediate	16.1 (4.5)	16.4 (4.0)	56.5 (19.3)	58.6 (19.5)	109.0 (48.6)	101.6 (41.0)	0.4 (0.2)	0.3 (0.3)	50.1 (14.3)	57.8 (16.9)	67.8 (25.9)	69.4 (23.8)	105.2 (34.5)	116.5 (47.7)	0.1 (0.2)	0.2 (0.2)
	Advanced	12.4 (2.0)	11.8 (1.9)	50.3 (13.0)	51.4 (17.6)	98.0 (29.9)	96.2 (34.3)	0.4 (0.2)	0.5 (0.2)	50.2 (13.8)	50.6 (16.3)	58.3 (14.9)	53.0 (19.2)	99.8 (33.5)	95.1 (33.9)	0.3 (0.2)	0.2 (0.2)
	Control	12.4 (2.0)	11.8 (1.9)	36.1 (9.1)	32.5 (9.8)	63.0 (21.2)	57.4 (21.1)	0.3 (0.2)	0.3 (0.3)	36.3 (6.8)	35.0 (8.7)	40.1 (08.6)	39.5 (14.6)	65.6 (18.1)	58.1 (22.3)	0.3 (0.2)	0.2 (0.2)
**NC-C**	Intermediate	15.4 (4.7)	16.0 (4.0)	56.0 (23.3)	53.1 (15.0)	110.4 (55.8)	106.9 (62.1)	0.3 (0.3)	0.4 (0.3)	58.4 (20.2)	48.1 (13.2)	64.6 (16.1)	59.8 (16.7)	104.1 (36.6)	94.7 (39.1)	0.3 (0.2)	0.2 (0.2)
	Advanced	14.2 (2.7)	14.7 (3.4)	47.7 (15.9)	49.0 (12.7)	93.7 (37.1)	91.6 (28.6)	0.3 (0.3)	0.4 (0.2)	47.2 (9.5)	42.5 (10.7)	50.3 (10.9)	51.0 (14.3)	82.2 (26.1)	83.7 (28.9)	0.3 (0.3)	0.2 (0.2)
	Control	11.5 (1.9)	11.6 (2.3)	34.8 (9.5)	32.1 (7.5)	59.7 (18.7)	55.8 (17.8)	0.3 (0.3)	0.4 (0.3)	37.3 (6.1)	32.1 (6.2)	40.7 (7.4)	37.7 (7.1)	62.3 (24.4)	57.3 (16.4)	0.4 (0.3)	0.3 (0.2)
**NC-NC**	Intermediate	20.1 (5.8)	17.6 (7.2)	65.5 (25.1)	61.5 (19.1)	120.0 (59.2)	124.6 (80.3)	0.3 (0.3)	0.4 (0.3)	52.1 (12.2)	52.8 (14.9)	74.9 (22.7)	66.5 (13.3)	127.0 (69.8)	112.0 (46.1)	0.3 (0.2)	0.2 (0.2)
	Advanced	16.9 (4.6)	17.8 (3.6)	54.4 (18.4)	56.3 (14.6)	107.7 (40.6)	107.4 (33.0)	0.4 (0.2)	0.4 (0.2)	46.4 (10.5)	48.3 (12.4)	55.7 (12.8)	59.5 (13.8)	93.7 (28.0)	96.7 (31.8)	0.3 (0.2)	0.3 (0.1)
	Control	11.9 (1.7)	12.4 (2.1)	36.7 (10.1)	33.9 (8.9)	63.4 (23.3)	61.5 (22.9)	0.3 (0.2)	0.3 (0.3)	35.3 (06.8)	33.9 (06.5)	37.1 (05.8)	37.2 (06.1)	62.3 (16.6)	64.9 (21.1)	0.3 (0.2)	0.3 (0.2)

FFD, First Fixation Duration; FPRT, First-Pass Reading Times; TRT, Total-Reading Times; RO, Regressions Out; C-C, Cognate-Cognate; NC-C, NonCognate-Cognate; C-NC, Cognate-NonCognate; NC-NC, NonCognate-NonCognate; HA, High Attachment; LA, Low Attachment.

Repeated-measures of variance (ANOVAs) were conducted for each of the remaining eye-tracking measures (FFD, FPRT, TRT, RO) both on the N1+N2 and N3 sentence regions, considering the reading performance of the L2 learner groups (intermediates and advanced) and of the English control group separately. Note that the critical comparisons at stake here are between the two L2 learning groups. The inclusion of the English control group as a third group in the analyses would not only highlight differences in reading performance that are not the main focus of this paper (e.g., English controls were faster than L2 learners in all the eye-movement measures considered), but, importantly, that option could contribute to mitigate the differences that between intermediates and advanced learners might be observed.

### English control group

For both regions, eye-tracking measures were analyzed using a 4 (Cognate status: C-C, C-NC, NC-C and NC-NC) x 2 (Disambiguation strategy: HA vs. LA) x 2 (List: 1 vs. 2) ANOVAs both on participants (*F*_1_) and items (*F*_2_) data. In the *F*_1_ analyses, Cognate status and Disambiguation strategy were considered as within-subject factors and List as a between-group factor, while in the *F*_2_ analyses Cognate status, Disambiguation strategy and List were considered as between-group factors. List was included both in the *F*_1_ and *F*_2_ analyses to remove the error of variance due to the existence of counterbalancing lists (see [60]). It is important to note that, although four lists of materials were constructed (see Materials section), for the purpose of these analyses, we only considered the two lists constructed according to the disambiguation strategy adopted in the RC, regardless of the fact that sentences were or not followed by a comprehension question. It is also worth noting that, although English native speakers had no prior knowledge of the EP language, which makes the distribution of the reading performance by cognate conditions somewhat artificial, we decided to introduce this factor in the analyses to further analyze if the materials used did not contain any confound that could inadvertently bias the results in any potential direction.

The Greenhouse-Geisser correction was applied for the analyses violating the assumption of sphericity, and for all the post hoc analyses performed the Bonferroni correction was applied. Nonsignificant effects (*p*s < .05) both in the *F*1 and *F*2 analyses are not reported, but results that reached statistical significance in the *F*1 analyses, even though not in the *F*2 analyses, are still presented. Although the absence of a statistically significant effect in the item analysis might suggest that the effect observed is not robust, it is important to note here that in the *F*2 analyses, the cognate status of the complex NP and the disambiguation strategy adopted in the RC were between-items variables, while in the *F*1 analyses they were within-subjects variables. As stated by Soares et al. (see [42]), this methodological difference has important implications in the results. In the *F*1 analyses the means were averaged over the items that each condition entailed and the nuisance variance caused by differences in the responses to the different items of the same condition was excluded. In the *F*2 analyses, however, the means were averaged over participants, hence making that the nuisance variance caused by differences in the responses to different items of the same condition cannot be excluded. Therefore, the variability in the responses in the item data is higher than in the participant data, which made any *F*2 effect much more difficult to be observed, particularly when using a small amount of stimuli (sentences), as in our case. The only way to increase the statistical power in the *F*2 analyses would be to increase the number of stimuli (sentences) per experimental condition, which was not possible due to the strict control that was imposed to the materials (see materials section). Thus, although caution is needed in the interpretation of the results that did not reach statistical significance in the *F*1 and *F*2 analyses, we opted to still present them and to explore their tendencies as in many other studies in sentence processing (e.g., [7], [15], [16], [19], [35], [42]). It is also worth noting that there are other sentence processing studies (e.g., [18], [21], [22]), that have opted to present only the results relative to the *F*1 analyses. Since the items used were matched in item-by-item basis (as in our case, see materials section), the *F*1 analysis is the most appropriate statistic because variance would be less within-conditions, making *F*2 too conservative as statistical tests of significance (see [61], [62] for details).

The ANOVAs conducted on the data from the English control group only showed a significant main effect of disambiguation in the N1+N2 region for FPRT, *F*_1_(1, 28) = 18.86, *MSE* = 27.15, *p* < .001, ɳ^2^_p_ = .40; *F*_2_(1, 80) = 2.82, *MSE* = 28.27, *p* = .097, ɳ^2^_p_ = .03, and in the N3 region for FFD, *F*_1_(1, 28) = 19.36, *MSE* = 28.31, *p* < .001, ɳ^2^_p_ = .41; *F*_2_(1, 80) = .772, *MSE* = 109.76, *p* = . 382, ɳ^2^_p_ = .01, and TRT, *F*_1_(1, 28) = 6.37, *MSE* = 1752.35, *p* = .018, ɳ^2^_p_ = .19; *F*_2_(1, 80) = .59, *MSE* = 317.30, *p* = .445, ɳ^2^_p_ = .01, measures. These results indicated that English native speakers were significantly faster reading the sentences disambiguated with an LA than with an HA strategy both in the N1+N2 and in the N3 sentence regions, as expected. Hence, the critical question was to analyze how the two L2 learner groups of English (intermediates and advanced), whose native language is EP (a language with an HA preference), would read the same sentences, and to what extent the cognate status of the complex NPs would affect their reading performance. The results obtained for the two L2 learning groups, in each of the four eye-tracking measures considered in the N1+N2 and N3 regions, are presented below.

### L2 learner groups

The statistical analyses conducted with the L2 learner groups parallel those conducted with the English control group, except that Proficiency (2: Intermediate vs. Advanced) was added as a between-subject factor in the *F*1 analyses and as a within-subject factor in the *F*2 analyses. The results obtained in both sentence regions (N1+N2 and N3) for each of the four eye-tracking measures, are presented below.

**FFD:** The ANOVAs showed a main effect of proficiency in the N3 region, *F*_1_(1, 56) = 9.48, *MSE* = 548.69, *p* = .003, ɳ^2^_p_ = .15; *F*_2_(1,80) = 45.48, *MSE* = 43.44, *p* < .001, ɳ^2^_p_ = .36, and also in the N1+N2 region, *F*_1_(1, 56) = 3.42, *MSE* = 64.02, *p* = .070, ɳ^2^_p_ = .06; *F*_2_(1,80) = 20.29, *MSE* = 4.12, *p* < .001, ɳ^2^_p_ = .20, although, in this case, the effect was only marginally significant in the by-participants analysis. This effect revealed longer FFDs for intermediate than for advanced learners. A main cognate status effect was also observed in the N1+N2, *F*_1_(3, 168) = 23.04, *MSE* = 11.01, *p* <. 001, ɳ^2^_p_ = .29; *F*_2_(3, 80) = 7.73, *MSE* = 12.72, *p* < .001, ɳ^2^_p_ = .23, and in the N3 region, *F*_1_(3, 168) = 4.82, *MSE* = 108.22, *p* = . 003, ɳ^2^_p_ = .08; *F*_2_(3, 80) = .39, *MSE* = 381.42, *p* = .764, ɳ^2^_p_ = .01. This effect showed that, in the N1+N2 region, participants took longer to read the complex NPs in the NC-NC condition than in all the other cognate conditions (all *p*s < .001). In the N3 region, the effect revealed that participants presented shorter FFDs in reading the critical word when it was preceded by a C-C than by a C-NC complex NP (*p* = .002). Moreover, a main disambiguation effect was also observed in the N3 region, *F*_1_(1, 56) = 9.96, *MSE* = 122.43, *p* = .003, ɳ^2^_p_ = .15; *F*_2_(1, 80) = 1.52, *MSE* = 381.42, *p* = .221, ɳ^2^_p_ = .02. This effect indicated that L2 learners were faster reading the critical word in the sentences disambiguated with an LA than with an HA strategy. A Cognate status x Disambiguation interaction was also observed in the N3 region, *F*_1_(3, 168) = 15.57, *MSE* = 76.23, *p* < .001, ɳ^2^_p_ = .22; *F*_2_(3, 80) = 1.71, *MSE* = 381.42, *p* = .171, ɳ^2^_p_ = .06. This effect revealed that the abovementioned LA advantage was restricted to the C-C (*p* < .001) and to the NC-C (*p* < .001) conditions. Furthermore, the pairwise comparisons indicated that in the sentences disambiguated with an LA strategy, participants showed shorter FFDs in reading the critical word in the C-C than both the C-NC (*p* < .001), and the NC-NC (*p* < .001) conditions.

**Fig 1:**
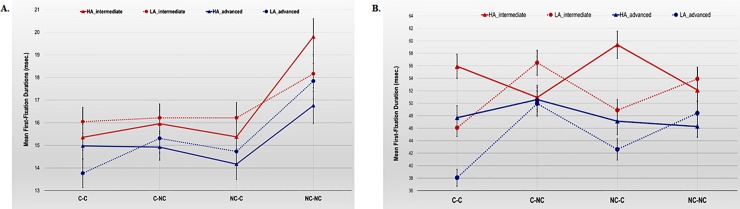
Means of First-Fixation Durations (ms) per character in the N1+N2 region (panel A) and in the N3 region (panel B) for intermediate and advanced L2 learners by cognate and disambiguation conditions. Error bars reflect the Standard Error Mean (SEM).

The three-way Proficiency x Cognate x Disambiguation interaction effect also reached statistical significance both in the N1+N2, *F*_1_(3, 168) = 2.99, *MSE* = 9.10, *p* = .032, ɳ^2^_p_ = .05; *F*_2_(3,80) = 2.40, *MSE* = 4.12, *p* = .074, ɳ^2^_p_ = .08, and in the N3 region, *F*_1_(3, 168) = 2.40, *MSE* = 76.23, *p* = .070, ɳ^2^_p_ = .04; *F*_2_(3, 80) = 3.11, *MSE* = 43.44, *p* = .031, ɳ^2^_p_ = .10, though, in the latter case, the effect was only marginally significant in the by-participants data. Fig 1 sets out the results obtained for each of the sentence regions to illustrate the effect.

As can be observed in Fig 1, the three-way interaction in the N1+N2 region (panel A) revealed that, although the differences between HA and LA reading times failed to reach statistical significance across cognate conditions in both groups of L2 learners (note, however, that intermediates tended to show longer FFDs in the HA sentences from the NC-NC condition than in the LA sentences from the same condition, *p* = .070), the results also indicated that participants from the intermediate group took longer to read the complex NP from the HA NC-NC condition than from any other HA cognate condition (all *p*s < .001). However, in the advanced learner group, these differences only reached statistical significance when the sentences from the HA NC-NC condition were compared with the sentences from the HA NC-C condition (*p* = .017). Furthermore, the analysis of the reading performance of the two groups in the LA sentences showed that, while advanced learners took longer to read the LA sentences from the NC-NC condition than from any other LA cognate condition (all *p*s < .001), in the intermediate learner group, these differences only reached statistical significance (marginal) when the LA NC-NC condition was compared with the LA C-C condition (*p* = .082). Hence, the NC-NC condition entails higher processing cost for both L2 learner groups, although it is interesting to note that for intermediates the differences only reached statistical significance when the HA NC-NC sentences were compared with all the other HA sentences, and for the advanced learner group when the LA NC-NC sentences were compared with all the other LA sentences.

In the N3 region (Fig 1, panel B), the three-way interaction revealed that intermediates showed shorter FFDs reading the critical word that disambiguated the sentences with an LA than with an HA strategy in the C-C (*p* < .001) and in the NC-C (*p* = .001) conditions, but shorter reading times in the sentences disambiguated with an HA than an LA strategy in the C-NC (*p* = .032) condition (in the NC-NC condition the differences were nonsignificant). The pairwise comparisons also revealed that, in the LA sentences, intermediates took longer to read the critical word in the LA C-NC than both the LA C-C (*p* < .001) and the LA NC-C (*p* = .049) conditions. The differences between LA C-C and LA NC-NC conditions also reached statistical significance (*p* = .009). However, in the sentences disambiguated with an HA strategy, intermediates took longer in reading the critical word in the HA NC-C condition than both the HA C-NC (*p* = .011) and the HA NC-NC (*p* = .045) conditions. In the C-C condition, advanced learners showed shorter FFDs in reading the sentences disambiguated with an LA than with an HA strategy (*p* < .001)—in the NC-C conditions these differences were nonsignificant. Moreover, the pairwise comparisons revealed that advanced learners showed shorter FFDs in the LA C-C than both the LA C-NC (*p* < .001) and the LA NC-NC (*p* < .001) conditions. The differences between the LA NC-C and the LA C-NC conditions approach significance (*p* = .060).

**FPRT**: The ANOVAs showed a main effect of proficiency both in the N1+N2, *F*_1_(1, 56) = 4.41, *MSE* = 1090.41, *p* = .040, ɳ^2^_p_ = .07; *F*_2_(1, 80) = 41.32, *MSE* = 39.97, *p* < .001, ɳ^2^_p_ = .34, and in the N3 regions, *F*_1_(1,56) = 15.41, *MSE* = 847.04, *p* < .001, ɳ^2^_p_ = .22; *F*_2_(1, 80) = 50.82, *MSE* = 107.33, *p* < .001, ɳ^2^_p_ = .39. This effect indicated that, in both regions, intermediates presented longer FPRTs than advanced learners, as in the previous reading time measure. A main cognate effect was also observed both in the N1+N2, *F*_1_(3, 130.01) = 12.30, *MSE* = 181.,83, *p* <. 001, ɳ^2^_p_ = .18; *F*_2_(3, 80) = 4.24, *MSE* = 158.43, *p* = .008, ɳ^2^_p_ = .14, and in the N3 regions, *F*_1_(3, 144.81) = 6.61, *MSE* = 266.86, *p* <. 001, ɳ^2^_p_ = .11; *F*_2_(3, 80) = 1.54, *MSE* = 389.27, *p* = .221, ɳ^2^_p_ = .06. In the N1+N2 region, the effect revealed that participants took longer to read the complex NPs in the NC-NC condition than in all the other cognate conditions (all *p*s < .001) in line with the previous results. In the N3 region, L2 learners were faster reading the critical word in the NC-C than both the NC-NC (*p* < .001) and the C-NC (*p* = .015) conditions. Moreover, L2 learners also showed shorter FPRTs in the C-C than in the NC-NC conditions (*p* = .035).

Additionally, the ANOVAs revealed a main disambiguation effect in the N3 region, *F*_1_(1, 56) = 13.91, *MSE* = 200.06, *p* < .001, ɳ^2^_p_ = .20; *F*_2_(1, 80) = 2.69, *MSE* = 389.27, *p* = .105, ɳ^2^_p_ = .03, indicating that participants were faster reading the critical word in the sentences disambiguated with an LA than with an HA strategy, as in the FFD measure. Furthermore, in the N3 region, a Cognate x Disambiguation interaction effect was also observed, *F*_1_(3, 168) = 3.65, *MSE* = 219.04, *p* = .014, ɳ^2^_p_ = .06; *F*_2_(3, 80) = 1.06, *MSE* = 389.27, *p* = .370, ɳ^2^_p_ = .04. This effect showed that the differences between LA and HA readings only reached statistical significance when the critical word was preceded by a C-C complex NP (*p* < .001), similarly to what was observed in the FFD measure (note, however, that the LA-HA difference previously observed in the NC-C conditions vanished). Moreover, the pairwise comparisons indicated that participants were faster in the LA C-C than both the LA C-NC (*p* < .001) and the LA NC-NC (*p* < .001) conditions. The differences between the LA NC-C and the LA NC-NC conditions also reached statistical significance (*p* = .007). In addition, participants also showed shorter FPRTs in the HA NC-C than in the HA NC-NC condition (*p* = .011).

**Fig 2:**
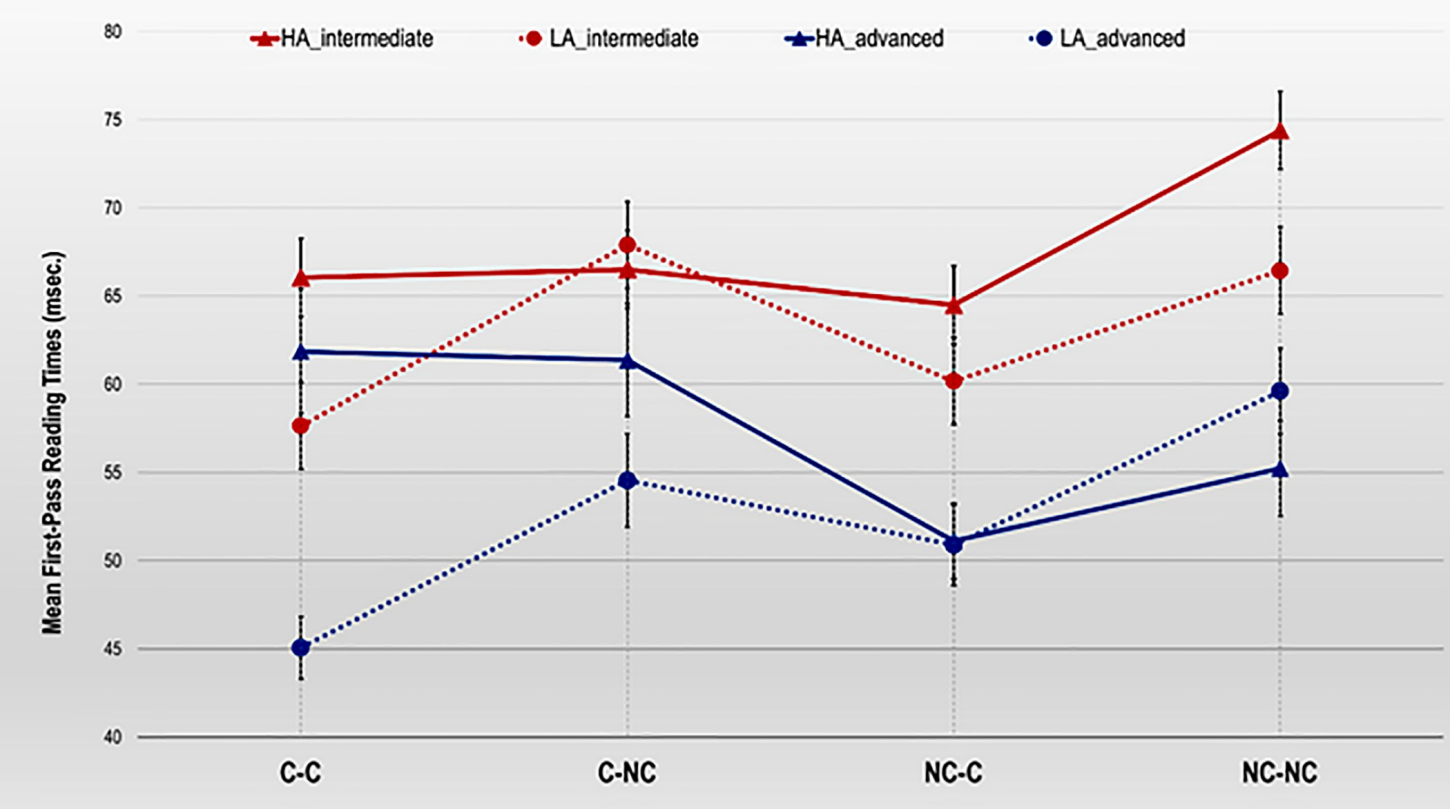
Means of First-Pass Reading Times (ms) per character in the N3 region for intermediate and advanced L2 learners by cognate and disambiguation conditions. Error bars reflect the Standard Error Mean (SEM).

Finally, the three-way Proficiency x Cognate status x Disambiguation interaction effect also reached statistical significance in the N3 region, *F*_1_(3, 168) = 3.46, *MSE* = 219.04, *p* = .018, ɳ^2^_p_ = .06; *F*_2_(3,80) = 3.40, *MSE* = 107.33, *p* = .022, ɳ^2^_p_ = .11. The interaction showed (see Fig 2) that intermediates presented shorter FPRTs in reading the critical word in the LA than in the HA sentences in the NC-NC (*p* = .020) and C-C (*p* = .061) conditions, though the differences in the latter case were marginally significant. Moreover, the pairwise comparisons revealed that intermediates showed shorter FPRTs in the LA C-C than both the LA C-NC (*p* = .005) and the LA NC-NC (*p* = .013) conditions. Finally, in the HA sentences, intermediates presented longer FPRTs in the NC-NC than in the NC-C (*p* = .011) conditions.

In the advanced learner group, the results indicated that participants showed shorter FPRTs in reading the critical word in the LA than in the HA sentences in the C-C (*p* < .001) and C-NC conditions (*p* = .085), though the differences in the latter were only marginally significant. Moreover, the pairwise comparisons also revealed that advanced learners presented shorter FPRTs in the LA C-C than both the LA NC-NC (*p* < .001) and the LA C-NC (*p* = .011) conditions. Furthermore, in the sentences disambiguated with an HA strategy, advanced learners tended to show shorter FPRTs in the NC-C condition than in the C-NC (*p* = .089) condition (the comparison between HA NC-C and HA C-C conditions was nonsignificant).

**TRT**: The ANOVAs showed a main effect of proficiency in the N3 region, *F*_1_(1, 56) = 6.02, *MSE* = 7333.27, *p* = .017, ɳ^2^_p_ = .10; *F*_2_(1, 80) = 49.68, *MSE* = 236.32, *p* < .001, ɳ^2^_p_ = .38, indicating that intermediates presented longer TRTs in reading the critical word than advanced learners, as observed in the previous measures (note however that the effect in the N1+N2 region did not reach statistical significance in this late temporal measure). A main cognate effect was also observed both in the N1+N2 *F*_1_(2.41, 135.05) = 18.22, *MSE* = 584.05, *p* < .001, ɳ^2^_p_ = .25; *F*_2_(1, 80) = 8.75, *MSE* = 358.42, *p* < .001, ɳ^2^_p_ = .25, and in the N3 regions, *F*_1_(3, 168) = 11.19, *MSE* = 617.56, *p* < .001, ɳ^2^_p_ = .17; *F*_2_(3, 80) = 2.12, *MSE* = 1309.40, *p* = .104, ɳ^2^_p_ = .07. In the N1+N2 region the effect revealed that participants took longer to read the complex NP in the sentences from the NC-NC condition than from all the other cognate conditions (all *p*s < .001). The results also indicated that participants showed longer TRTs in the C-NC than in the C-C (*p* = .029) condition. In the N3 region, the effect indicated that L2 learners showed longer TRTs in the NC-NC than both the C-C (*p* = .014) and the NC-C (*p* < .001) conditions. Additionally, participants also took longer to read the critical word in the C-NC than both the C-C (*p* = .030) and the NC-C (*p* = .001) conditions.

A main disambiguation effect was also observed in the N3 region, *F*_1_(1, 56 = 21.18, *MSE* = 344.23, *p* < .001, ɳ^2^_p_ = .27; *F*_2_(1, 80) = 1.94, *MSE* = 1309.40, *p* = .167, ɳ^2^_p_ = .02. As in the previous reading time measures, this effect showed that L2 learners were faster in reading the critical word when the sentences were disambiguated with an LA than with an HA strategy. However, as in the FPRT measure, the Cognate x Disambiguation interaction effect observed in this sentence region, *F*_1_(3, 168) = 7.60, *MSE* = 571.64, *p* < .001, ɳ^2^_p_ = .12; *F*_2_(3, 80) = 1.69, *MSE* = 1309.40, *p* = .177, ɳ^2^_p_ = .06, revealed that this LA advantage was restricted, once again, to the C-C (*p* < .001) condition. Moreover, the pairwise comparisons showed that participants were faster reading the critical word in the LA C-C condition than both the LA C-NC (*p* < .001) and the LA NC-NC (*p* < .001) conditions. Participants were also faster reading the critical word in the LA NC-C than both the LA C-NC (*p* = .003) and the LA NC-NC (*p* = .001) conditions; and also in the LA NC-C than in the LA NC-NC condition (*p* = .001). In the HA sentences, the pairwise comparisons also revealed that participants were also faster reading the critical word in the in the NC-C than in the NC-NC conditions (*p* = .006). The differences between the HA NC-C and the HA C-C conditions approach significance (*p* = .074).

**Fig 3:**
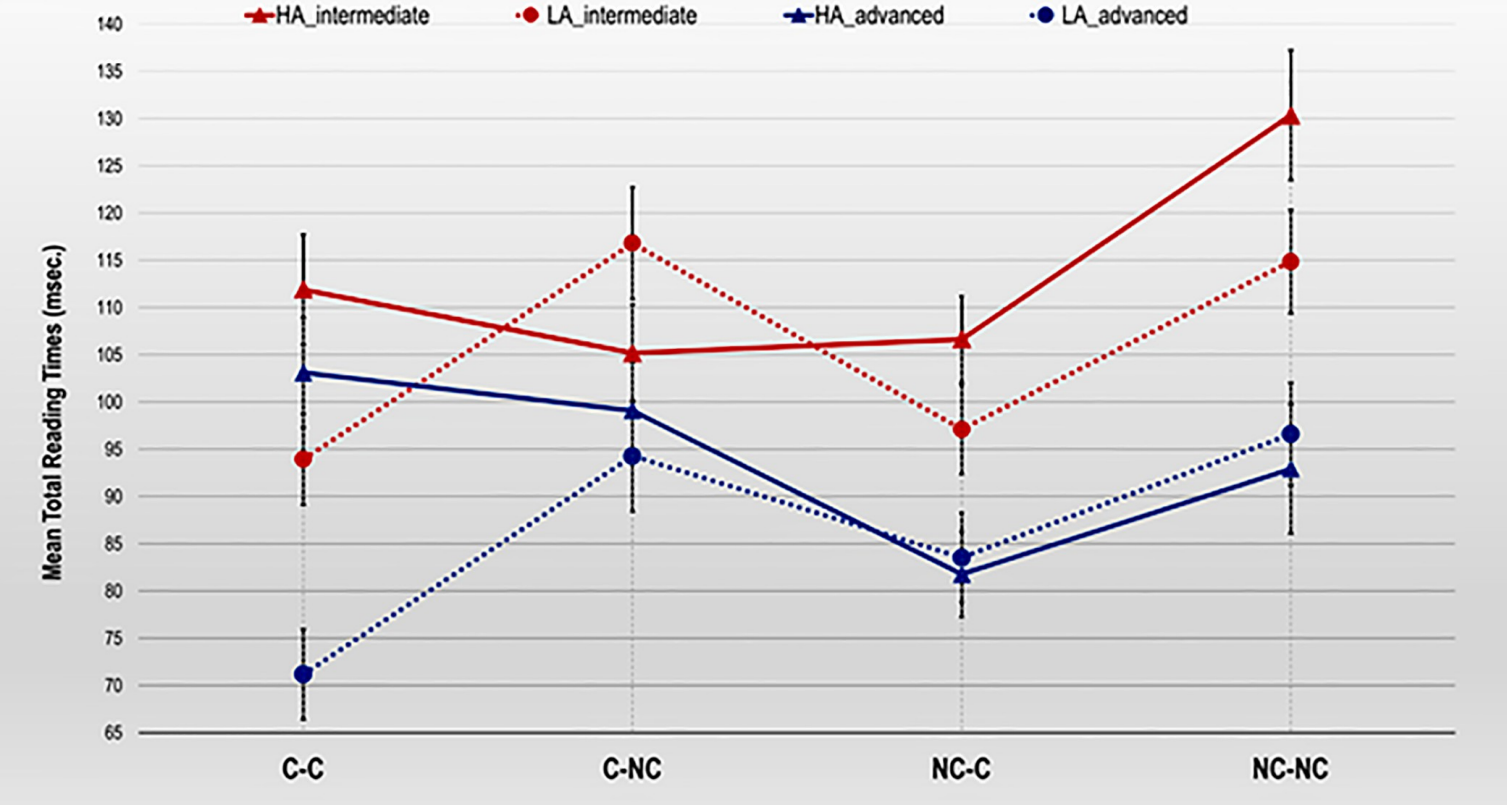
Means of Total Reading Times (ms) per character in the N3 region for intermediate and advanced L2 learners by cognate and disambiguation conditions. Error bars reflect the Standard Error Mean (SEM).

Finally, the three-way Proficiency x Cognate status x Disambiguation interaction effect reached statistical significance in the N3 region, *F*_1_(3, 168) = 4.15, *MSE* = 571.64, *p* = .007, ɳ^2^_p_ = .07; *F*_2_(3, 80) = 4.85, *MSE* = 236.32, *p* = .004, ɳ^2^_p_ = .15. This effect revealed, as illustrated in Fig 3, that intermediates showed shorter TRTs in reading the critical word in the sentences disambiguated with an LA than with an HA strategy in all the cognate conditions (all *p*s < .05), except in the C-NC condition, in which the HA sentences tended to be read faster than the LA sentences (*p* = .073). The pairwise comparisons also revealed that intermediates showed shorter TRTs in the LA C-C than both the LA C-NC (*p* < .001) and the LA NC-NC (*p* = .005) conditions. The differences between the LA NC-C and both the LA C-NC (*p* < .001) and the LA NC-NC (*p* = .005) conditions were also statistically significant. In the sentences disambiguated with an HA strategy, intermediates showed longer TRTs in the HA NC-NC than both the HA C-NC (*p* = .010) and the HA NC-C (*p* = .010) conditions.

In the advanced learner group, the differences between LA-HA readings only reached statistical significance in the C-C condition, in which participants showed faster TRTs in reading the critical word in the sentences disambiguated with an LA than with an HA strategy (*p* < .001). Moreover, the pairwise comparisons also revealed that participants were faster reading the critical word in the LA C-C condition than both in the LA C-NC (*p* < .001) and the LA NC-NC (*p* < .001) conditions. The differences between the LA NC-C and the LA NC-NC condition reached statistical significance (*p* = .003) although the differences between the LA C-C and the LA NC-C conditions were only marginally significant (*p* = .089). Finally, the results also indicated that advanced learners showed shorter TRTs in the HA NC-C than both the HA C-C (*p* = .028) and the HA C-NC (*p* = .014) conditions.

**RO**: The ANOVAs showed a significant main effect of disambiguation in the N1+N2 region, *F*_1_(1, 56) = 4.19, *MSE* = .040, *p* = .045, ɳ^2^_p_ = .07; *F*_2_(1, 80) = .30, *MSE* = .032, *p* = .586, ɳ^2^_p_ = .004,. This effect revealed that participants made more ROs in the sentences disambiguated with an LA (0.40) than with an HA strategy (0.37). In the N3 region, the results indicated a main effect of cognate, *F*_1_(3, 168) = 5.05, *MSE* = .030, *p* = .002, ɳ^2^_p_ = .08; *F*_2_(3, 80) = 2.02, *MSE* = .019, *p* = .118, ɳ^2^_p_ = .07 indicating that L2 learners made more ROs when the critical word was preceded by a NC-C than by a C-NC (*p* = .022) complex NP.

## Discussion

The present study aimed to examine how the lexical and syntactic levels of processing interact during L2 sentence processing and how those interactions were modulated by L2 proficiency, by using an online technique (eye-tracking) that is highly sensitive to the lexical and syntactic processes involved in sentence reading. To that purpose, native-speakers of EP learning English as L2 at intermediate and advanced levels of proficiency were asked to silently read temporally ambiguous L2 RC sentences while their eye-movements were monitored. This grammatical structure was chosen because previous studies had shown that EP and English exhibit opposite preferences in the resolution of this syntactic ambiguity (HA and LA, respectively), hence allowing us to use the HA preference as a marker of L1 syntactic interference. Additionally, the embedding of cognates and noncognates in the complex NP of this grammatical structure, was also used to further analyze how the lexical co-activation of both languages would also affect the syntactic representations of the non-target (L1) language, hence increasing the level of cross-language competition for RC attachment. It is also important to note that this paper followed another one, where Soares et al. ([42)] examine the same research questions by using an offline (sentence completion) task, which did not allow the authors to explain why EP (L1)-English (L2) bilinguals showed more HA completions in the NC-NC than in the C-C conditions, and also in the NC-C than in the C-NC conditions. The use of the eye-tracking technique in this study aimed to directly address these issues and to disentangle the two hypotheses (the cognitive load hypothesis and the resource capacity hypothesis) advanced by Soares et al. ([42]) to explain those results.

The findings obtained for the two sentence regions analyzed (N1+N2 and the N3) provide evidence for the lexical co-activation of both languages during L2 RC sentence processing, and, importantly, that the cognate status of the words embedded in the complex NP of the RC structure affected the extent to which L1 RC preferences were activated, thus suggesting that the lexical and syntactic levels of representation interact during L2 sentence processing in a bilingual reading system that is not only highly interactive within each level of processing, but, critically, across levels of representation, as predicted. Specifically, the results indicated shorter reading times when the complex NP of the RC sentences (i.e., the N1+N2 region) entailed cognates than noncognates, which extends previous findings of a cognate facilitative effect in L2 sentence processing (e.g., [12], [14], [15], [16], [17], [18], [19], [20]) to another (RC) grammatical structure. Moreover, the cognate facilitative effect observed in the N1+N2 region was not only noticed in early reading time measures (FFD, FPRT, indexing processes of lexical access and syntactic parsing), as observed in previous eye-tracking studies (e.g., [14], [16], [20], [41]), or in later reading time measures (TRT, indexing processes of language integration)—see for example [17], [19], [20], see also [15] but only for identical cognate, but, importantly, in both. These findings support the view that the embedding of cognates in the complex NP of the RC structure induce a longstanding cognate effect on L2 sentence processing.

Furthermore, it is also worth noting that the facilitative cognate effect observed in our data was not restricted to the N1+N2 region (the region in which cognate words were actually embedded), but was also observed in the N3 region (the region that entails the critical word that disambiguated the sentences towards one of its potential directions). These results suggest that the cognate advantage observed in the L2 RC sentence processing, not only extends previous findings on cognate facilitation in sentence processing to another grammatical structure, as abovementioned, but also demonstrates that the lexical co-activation generated by the embedding of cognates (and noncognates) in the complex NPs was strong enough as to affect the reading performance in other sentence regions. The use of two cognates (and/or noncognates) in the complex NPs, rather than one single cognate, as in most L2 sentence studies conducted do far (e.g., [14], [15], [16], [20], [19], [41]), might also account for these results. Indeed, using more than one cognate could have simply contributed to strongly co-activate the L1 lexicon and to strengthen the cognate effect observed in other sentence regions.

Nevertheless, cognate facilitative effects were found in the N3 region not only when the complex NP entailed two cognates (C-C condition), but also when it entailed a single cognate, particularly when the cognate appeared at the second position of the complex NP, giving rise to a cognate position effect, as previously observed by Soares et al. ([42]) in their sentence completion study. This effect, observed both in early and late reading time measures, showed that participants were significantly faster reading the critical word in the RC when the last word of the complex NP was a cognate than when it was a noncognate. However, the fact that participants were faster reading the critical word in the NC-C than in the C-NC condition also entailed a cost, as participants made more regressive movements (ROs) in the NC-C condition than in the C-NC condition, which was probably due to a trade-off effect: as participants were faster in the NC-C than in the C-NC condition, this might also cause that they needed to look back more times for reading comprehension.

The cognate position effect observed in our data can be explained if we consider, on one hand, that noncognates are harder to process than cognates (e.g., [14], [15], [16], [17], [19], [20], [41]), and, on the other hand, that L2 learners showed a ‘delayed’ L2 sentence processing (e.g., [43], [44], [45], see also Table 4 for native/non-native reading time comparisons), which might have produced a sort of spillover effect when noncognates appeared at the second position in the complex NP. Indeed, in that situation, the higher cost in the processing of noncognates might have simply spread out to the next sentence region, thus explaining longer reading times in the critical word in the RC was preceded by a noncognate than by a cognate. In support to this explanation is also the fact that neither the differences between the NC-C and the C-C conditions, nor the differences between the C-NC and the NC-NC conditions reached statistical significance, thus suggesting that the cognate effect observed in the N3 region is driven by the cognate status of the last constituent of the complex NP. Note that these results cannot be attributed to some artifact in the materials, since the English control group did not show any signs of a cognate effect when reading the same sentences in any of the measures and sentence regions considered. The only significant effect observed in the control group was a main effect of disambiguation, showing in the N1+N2 and N3 regions shorter reading times in the sentences disambiguated with an LA than with an HA strategy, as observed in previous studies with English native-speakers (e.g., [3], [4], [6], [7], [42]), thus providing further evidence to assume the LA RC preference as an ‘English native way’ of reading temporally ambiguous RC sentences.

Importantly, the analysis of the reading performance of the two L2 learner groups demonstrated that the lexical co-activation generated by the embedding of cognates (and noncognates) in the complex NP of the RC structure impacted the way L2 learners processed and comprehended L2 RC sentences, thus suggesting that the lexical and the syntactic levels of representations interact during L2 sentence processing, as predicted. Indeed, although the L2 learners were significantly faster reading the sentences disambiguated with an LA than with an HA strategy in all reading time measures from the N3 region, the differences between the LA and the HA only reached statistical significance in the C-C and NC-C conditions in the early reading time measures, though restricted to the C-C condition in later reading time measures. The LA advantage observed in the C-C and NC-C conditions in early (FFD) reading time measures clearly suggests that presenting a cognate at the second position of the complex NP induces lower L1 RC syntax interference, thus explaining longer reading times in the HA than in the LA sentences from these conditions. Conversely, in the C-NC and NC-NC conditions, the absence of any statistically significant difference between HA-LA readings suggests that the L1 RC syntactic interference was stronger, thus making participants to read the LA sentences as fast as the HA sentences from these conditions. Bear in mind that a stronger L1 RC syntactic activation means that both strategies were equally available for reading, thus making that HA-LA reading times were not statistically different. Note, however, that, in the N1+N2 region, the effect of disambiguation observed in the RO measure showed an higher reading cost for the LA than for the HA sentences, hence suggesting that at this point of sentence processing the L1 RC preference seems to be automatically activated. These results are interesting and consistent with the previous findings reported by Soares et al. ([42]), tough they additionally show that the use of cognates facilitated and not hampered a more ‘English native-like way’ of L2 RC sentence processing, hence providing support to the resource capacity hypothesis advanced in the Introduction. Indeed, if we assume that noncognates are harder to process than cognates, we might also anticipate that, under conditions of increased lexical demands, the processor might not release the resources necessary to strongly inhibit L1 RC syntactic preferences, thus making that L1 RC syntactic interference to be more noticeable for noncognates than for cognates, as advanced by Hopp [[41]).

However, if the capacity resource hypothesis can account for the results observed in the NC-NC and in the C-C conditions, it cannot explain why stronger L1 RC syntactic interference was observed in the C-NC than in the NC-C condition, i.e., for the cognate position effect observed in the N3 region in the FFD measure. Indeed, since the C-NC and the NC-C represent conditions of similar lexical demands (as both entail one cognate and one noncognate), differences between them were not expected according to the capacity resource hypothesis, as mentioned. Yet, the results indicated that in the NC-C condition participants presented shorter reading times in the LA than in the HA sentences, whereas in the C-NC condition, the differences between the HA-LA readings did not reach statistical significance. Additionally, it should be also noted that in the NC-C condition participants revealed less regressive eye-movements in the LA than in the HA sentences, which provides further support for the idea that LA sentences were not only processed faster than the HA sentences but were also better comprehended. Taken together, these findings suggest stronger L1 RC syntactic co-activation when the cognate occupies the L1 RC preferential position (i.e., in the C-NC) than when it occupies the non-preferential position (i.e., in the NC-C position), which seems to be in accordance with the predictions of the cognitive load hypothesis. Alternatively, it is also possible, in line with the cognitive load hypothesis, that, since L2 sentence processing is more demanding than L1 sentence processing (e.g., [41], [43], [44], [51]), the L2 learners in our study might be simply more prone to establish local (LA) than non-local (HA) attachments, thus being more sensitive to the lexical properties of the last processed item. Under this assumption, the C-NC condition would represent a higher processing load than the NC-C condition, thus explaining the cognate position effect observed.

Nonetheless, it is also important to note that the results obtained showed that the impact of the cognate composition of the NPs on L2 RC syntactic processing affected intermediate learners more strongly than the advanced learners. Indeed, the three-way interaction observed in all the reading time measures in the N3 region, showed that, at lower levels of proficiency, the L2 RC preferences were modulated by the position in which the cognate appears in the complex NP. Specifically, the results showed that, in early (FFD) reading time measures, intermediates were significantly faster in reading the critical word that disambiguated the sentences with an LA than with an HA strategy in the C-C and NC-C conditions. However, in the C-NC condition, intermediates were significantly faster in reading the critical word in the sentences disambiguated with an HA than with an LA strategy (the HA-LA differences in the NC-NC condition did not reach statistical significance). These findings suggest that the ease with which intermediates read and comprehend the L2 RC sentences seems to depend on the match between the position at which the cognate appears in the complex NP (first vs. second position) and the attachment strategy adopted in the RC (HA vs. LA). On the FPRT and TRT measures, however, the LA advantage was restricted to the C-C condition. Interestingly, on the late (TRT) reading time measure, the LA-HA differences also reached statistical significance in the NC-NC condition, showing that intermediates took longer to read the sentences disambiguated with an HA than with an LA strategy, in line with the results observed in the N1+N2 region. This finding suggests that at conditions of higher processing load (NC-NC), intermediates read faster the sentences disambiguated with an LA than with an HA strategy, probably because the former are cognitively less consuming than the latter, thus stimulating the use of a recency strategy (LA).

Conversely, the reading performance of advanced learners was not so strongly affected by the cognate composition of the complex NPs. Indeed, in all the cognate conditions, advanced learners read the LA sentences as fast as the HA sentences, except in the C-C condition, in which they were significantly faster reading the LA than the HA sentences. These results are in line with our predictions and show that L2 proficiency matters, not only because advanced learners were faster than intermediate learners, as observed in several eye-tracking studies (e.g., [14], [16], [20], [19], [25], [41]), but, essentially, because they demonstrate that the bilingual reading system is highly dynamic, with the level of proficiency modulating the nature of the lexico-syntactic interactions established during L2 sentence processing in the bilingual mind. Intermediate learners seem to show a more ‘lexicalized L2 RC syntactic processing’, as their reading performance was clearly affected by the position at which the cognate appeared in the complex NP, while advanced learners seem to go beyond the lexical properties of the items and to use the structural information provided by the sentence more efficiently. The extent to which this reflects an L2 native-like way of processing is controversial. Note that, contrary to the English control group, which showed a global LA advantage in the RC sentence readings, the L2 learners from the advanced group did not show any RC attachment preference in reading the same sentences, except in the C-C condition, as mentioned. Thus, rather than showing a sentence processing that mimics the one observed in the English control group, advanced learners seem to show a qualitatively distinct way of L2 RC processing. Knowing and mastering an L2 seem to have changed profoundly the way L2 learners process and comprehend L2 sentences, in a reading system that seems to be much more complex, enriched, and flexible, with both strategies being equally available to guide L2 reading comprehension.

Moreover, it is important to emphasize that these results also suggest that, as L2 proficiency increases, the L1 syntax interference becomes stronger and not weaker, as hypothesized. Indeed, against our predictions and to the claims of the CM ([29], [30]), our results indicate that, as proficiency increases, the L1 RC syntax interference becomes stronger in a syntactic parser that appears to work in a progressively more integrated and nonselective way, as recently suggested by L2 syntactic priming studies (e.g., [34], [40]). Note, however, that, if advanced learners seem to experience higher levels of syntactic competition for RC attachment, their reading performance was not adversely affected by that, as reflected by both faster reading times than intermediates in all reading time measures and by the absence of any significant cost in the RO measure. The more efficient executive control strategies that high-proficiency learners tend to present (e.g., [17]) might also explain why the higher cross-language activation of syntax from languages that showed different RC attachment preferences as EP and English did not cause detrimental effects in the bilingual reading system.

Altogether, these results provide further evidence for a shared account of syntax in bilinguals, and also for a developmental view of syntax in bilinguals, which shifts from having separate and item-specific syntactic representations for structures that are similar across-languages (even without presenting the same syntactic preferences), to shared and more abstract syntactic representations, as recently suggested by Bernolet et al. ([40]) and others (e.g., [34]). At low levels of proficiency, the syntactic preferences of each language seem to be represented separately, and the use of one or another seems to be determined by the lexical properties of the items used in the NPs. However, at higher levels of proficiency both strategies seem to be equally available for reading regardless of the lexical composition of the NPs. This suggests that, as L2 learners become more proficient, the RC syntactic representations of each language are merged into a single representation that is shared across-languages. Thus, highly proficient bilinguals were able to read HA and LA RC sentences in their L2 equally fast and without any apparent cost in a parser that seems to act in a more flexible and less language-dependent way. Nevertheless, it remains unclear whether this way of functioning would be also observed when L2 learners would read the same RC sentences in their L1. If the RC syntactic representations are really shared across-languages and both languages use the same syntactic representations (at least at higher levels of proficiency), the same pattern of results would be expected regardless of the language in use. Future research should address these issues.

## Supporting information

S1 Dataset Dataset from native and second language learner groups. (XLSX)

S1 Fig **Means of First-Fixation Durations (ms) per character in the N1+N2 region (panel A) and in the N3 region (panel B) for intermediate and advanced L2 learners by cognate and disambiguation conditions.** Error bars reflect the Standard Error Mean (SEM).
(TIF)

S2 Fig Means of First-Pass Reading Times (ms) per character in the N3 region for intermediate and advanced L2 learners by cognate and disambiguation conditions. Error bars reflect the Standard Error Mean (SEM).
(TIF)

S3 Fig Means of Total Reading Times (ms) per character in the N3 region for intermediate and advanced L2 learners by cognate and disambiguation conditions. Error bars reflect the Standard Error Mean (SEM).
(TIF)

S1 Table Means and Standard Deviations (in brackets) for the subjective ratings of reading, writing, speaking, and listening skills in the two groups of L2 learners. (PDF)

S2 Table Means and Standard Deviations (in brackets) of the psycholinguistic characteristics of N1, N2, and N3 words used in the experimental sentence in the four cognate conditions. (PDF)

S3 Table Means and Standard Deviations (in brackets) of the plausibility ratings obtained for the experimental sentences in English and EP by cognate condition. (PDF)

S4 Table Means and Standard Deviations (in brackets) of the FFD, FPRT, and TRT measures, and of the proportions of RO in the complex NP region (N1+N2) and in the critical word that disambiguates the sentence (N3 region) by experimental condition and participant group. (PDF)
