# Supplementary figures and images for: Lexico-syntactic interactions during the processing of temporally ambiguous L2 relative clauses: An eye-tracking study with intermediate and advanced Portuguese-English bilinguals
Source: PLoS One. 2019 May 29;14(5):e0216779. doi: 10.1371/journal.pone.0216779 (PMC6541246; doi:10.1371/journal.pone.0216779)

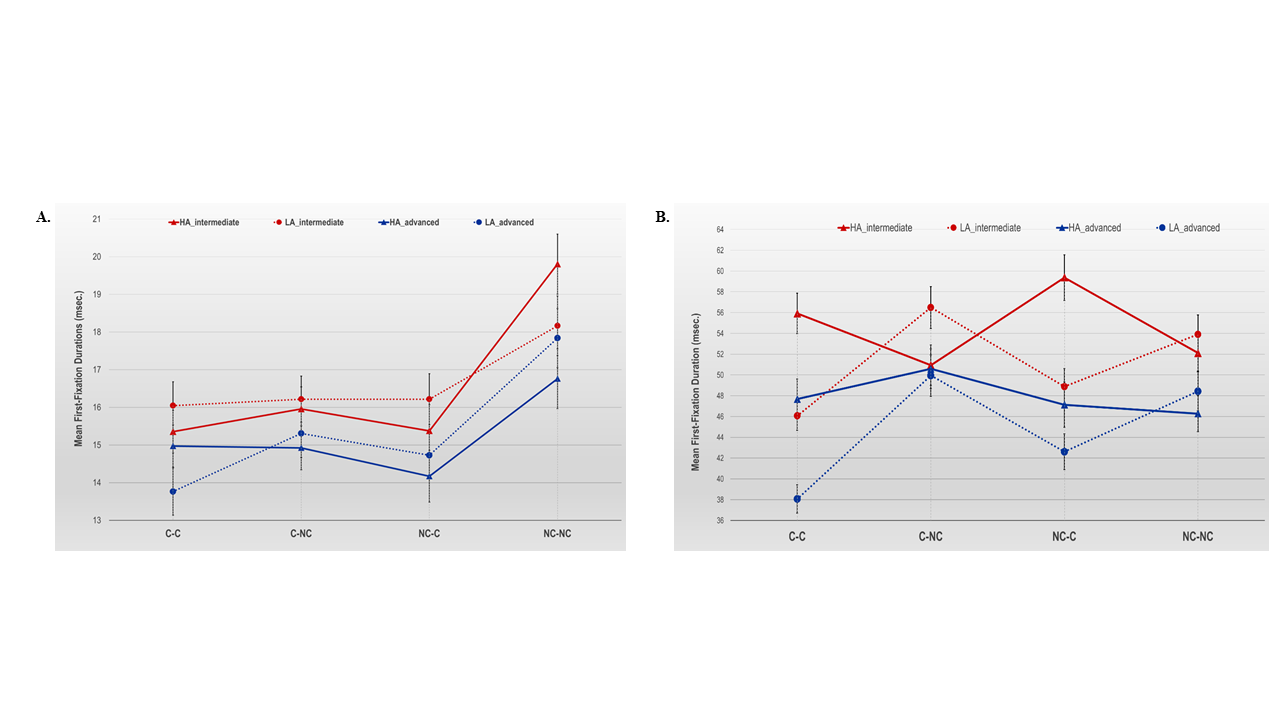

Supplement: S1 Fig — Means of First-Fixation Durations (ms) per character in the N1+N2 region (panel A) and in the N3 region (panel B) for intermediate and advanced L2 learners by cognate and disambiguation conditions. Error bars reflect the Standard Error Mean (SEM). (TIF) [file pone.0216779.s002.TIF]

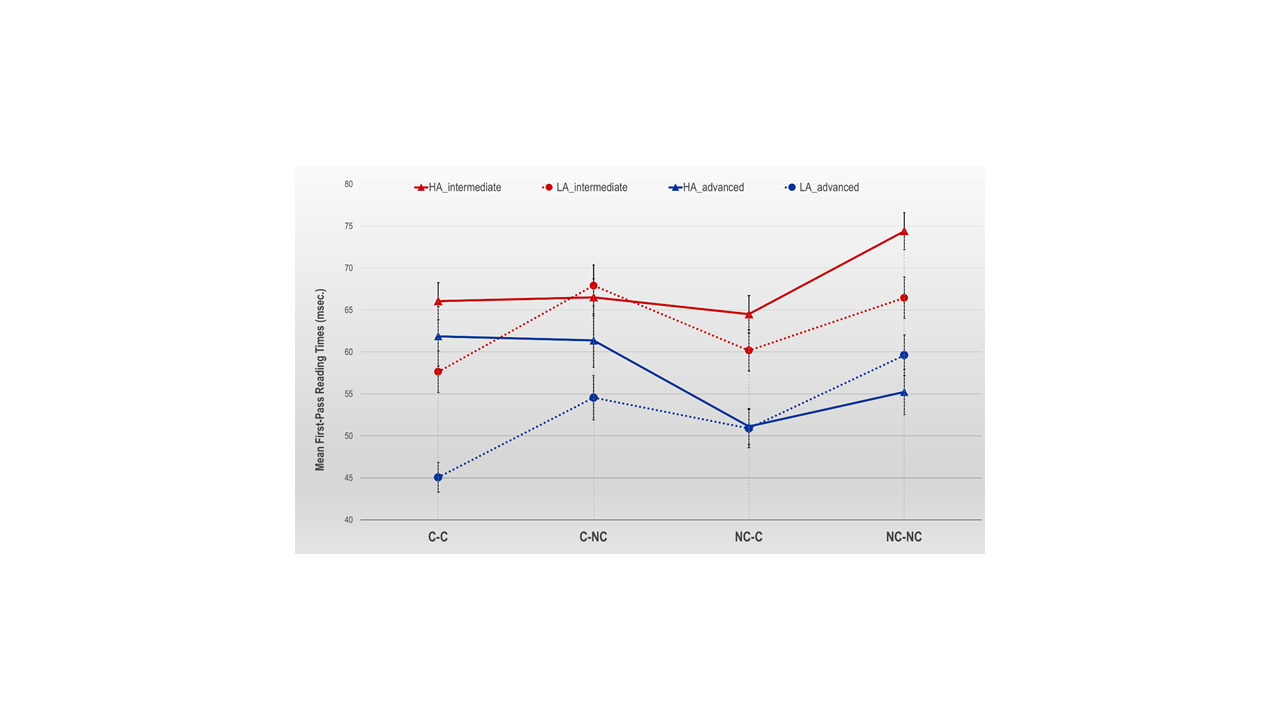

Supplement: S2 Fig — Error bars reflect the Standard Error Mean (SEM). (TIF) [file pone.0216779.s003.TIF]

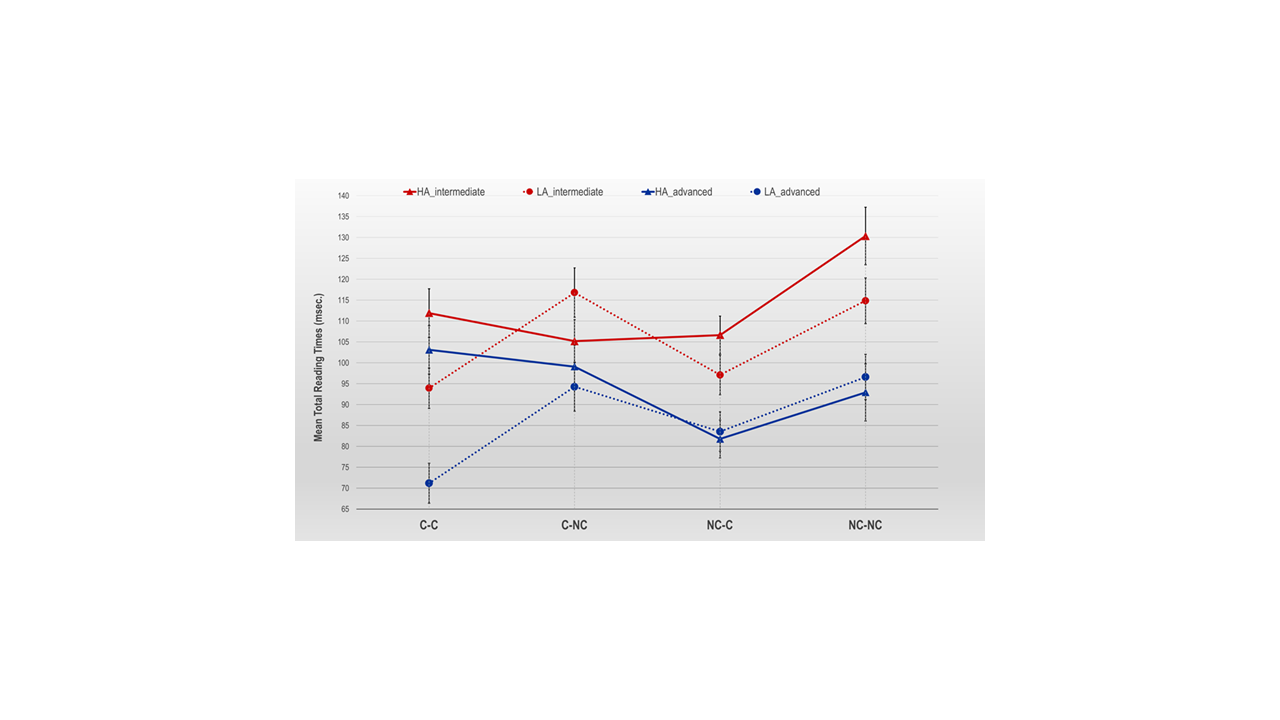

Supplement: S3 Fig — Error bars reflect the Standard Error Mean (SEM). (TIF) [file pone.0216779.s004.TIF]
