# Supplementary material for: Lexico-syntactic interactions during the processing of temporally ambiguous L2 relative clauses: An eye-tracking study with intermediate and advanced Portuguese-English bilinguals
Source: PLoS One. 2019 May 29;14(5):e0216779. doi: 10.1371/journal.pone.0216779 (PMC6541246; doi:10.1371/journal.pone.0216779)
Supplement: S1 Table — (PDF) [file pone.0216779.s005.pdf]

Table 1. Means and Standard Deviations (in brackets) for the subjective ratings of reading, writing, speaking, and listening skills in the two groups of L2 learners.

L2 groups	Reading	Writing	Speaking	Listening
Intermediate	5.52 (0.85)	4.89 (1.16)	5.04 (1.29)	5.44 (1.09)
Advanced	6.10 (0.71)	5.37 (0.93)	5.60 (0.97)	6.03 (0.89)

7-point Likert scale ranging from 1 “very poor” to 7 “native-like”.
