# Supplementary material for: Lexico-syntactic interactions during the processing of temporally ambiguous L2 relative clauses: An eye-tracking study with intermediate and advanced Portuguese-English bilinguals
Source: PLoS One. 2019 May 29;14(5):e0216779. doi: 10.1371/journal.pone.0216779 (PMC6541246; doi:10.1371/journal.pone.0216779)
Supplement: S2 Table — (PDF) [file pone.0216779.s006.pdf]

Table 2. **Means and Standard Deviations (in brackets) of the psycholinguistic characteristics of N1, N2, and N3 words used in the experimental sentence in the four cognate conditions.**

Sentence condition	Psycholinguistic characteristics	N1	N2	$N3_{HA}$	$N3_{LA}$
**C-C**	**Length English**	7.1 (1.7)	6.4 (1.2)	6.1 (2.3)	6.7 (1.4)
	**Length EP**	7.5 (1.8)	6.8 (2.0)	7.8 (2.2)	8.3 (2.0)
	**Frequency English**	3.9 (0.9)	4.1 (0.4)	4.1 (0.6)	4.3 (0.6)
	**Frequency EP**	4.1 (0.7)	4.1 (0.6)	3.8 (0.7)	3.7 (1.1)
	**Orthographic overlap**	0.6 (0.2)	0.7 (0.2)	0.2 (0.1)	0.2 (0.1)
**C-NC**	**Length English**	6.4 (3.3)	6.2 (1.8)	5.8 (1.7)	5.8 (1.6)
	**Length EP**	6.9 (3.3)	8.0 (2.2)	7.6 (2.5)	7.3 (2.3)
	**Frequency English**	4.4 (0.7)	4.3 (0.5)	4.1 (1.1)	4.2 (0.7)
	**Frequency EP**	4.3 (0.7)	4.4 (0.8)	3.2 (1.0)	3.8 (0.9)
	**Orthographic overlap**	0.6 (0.1)	0.2 (0.1)	0.2 (0.1)	0.2 (0.1)
**NC-C**	**Length English**	6.1 (1.2)	7.3 (1.7)	5.3 (1.1)	6.8 (2.1)
	**Length EP**	5.9 (1.7)	7.4 (2.4)	6.8 (2.5)	7.6 (2.1)
	**Frequency English**	4.2 (0.5)	4.4 (0.5)	4.6 (0.7)	4.0 (0.8)
	**Frequency EP**	4.3 (0.5)	4.4 (0.3)	4.2 (0.7)	3.8 (0.7)
	**Orthographic overlap**	0.2 (0.1)	0.7 (0.1)	0.1 (0.1)	0.2 (0.1)
**NC-NC**	**Length English**	5.0 (2.1)	5.6 (1.8)	6.4 (1.6)	5.8 (1.8)
	**Length EP**	5.6 (2.0)	5.9 (1.6)	6.2 (1.5)	6.4 (1.2)
	**Frequency English**	4.3 (0.6)	4.6 (0.5)	3.8 (0.85)	4.2 (0.71)
	**Frequency EP**	4.1 (1.2)	4.5 (0.5)	3.8 (1.1)	4.1 (1.0)
	**Orthographic overlap**	0.1 (0.1)	.09 (0.1)	0.2 (0.1)	0.2 (0.2)

C-C, Cognate-Cognate; NC-C, NonCognate-Cognate; C-NC, Cognate-NonCognate; NC-NC, NonCognate-NonCognate; N1, first-noun of the complex noun phrase; N2, second-noun of the complex noun phrase; $N3_{HA}$, critical word that disambiguates the sentence with a high attachment strategy; $N3_{LA}$, critical word that disambiguates the sentence with a low attachment strategy; EP, European Portuguese.
