# Supplementary material for: Lexico-syntactic interactions during the processing of temporally ambiguous L2 relative clauses: An eye-tracking study with intermediate and advanced Portuguese-English bilinguals
Source: PLoS One. 2019 May 29;14(5):e0216779. doi: 10.1371/journal.pone.0216779 (PMC6541246; doi:10.1371/journal.pone.0216779)
Supplement: S3 Table — (PDF) [file pone.0216779.s007.pdf]

Table 3. **Means and Standard Deviations (in brackets) of the plausibility ratings obtained for the experimental sentences in English and EP by cognate condition.**

Language	Cognate Condition	HA	LA
English	**C-C**	4.6 (0.3)	4.7 (0.3)
	**C-NC**	4.7 (0.3)	4.7 (0.2)
	**NC-C**	4.6 (0.2)	4.6 (0.4)
	**NC-NC**	4.5 (0.5)	4.7 (0.2)
EP	**C-C**	4.8 (0.1)	4.8 (0.2)
	**C-NC**	4.8 (0.2)	4.8 (0.1)
	**NC-C**	4.8 (0.2)	4.7 (0.3)
	**NC-NC**	4.8 (0.2)	4.9 (0.1)

5-point Likert scale ranging from 1 “not plausible” to 5 “very plausible”; C-C, Cognate-Cognate; NC-C, NonCognate-Cognate; C-NC, Cognate-NonCognate; NC-NC, NonCognate-NonCognate.
