# Supplementary material for: Lexico-syntactic interactions during the processing of temporally ambiguous L2 relative clauses: An eye-tracking study with intermediate and advanced Portuguese-English bilinguals
Source: PLoS One. 2019 May 29;14(5):e0216779. doi: 10.1371/journal.pone.0216779 (PMC6541246; doi:10.1371/journal.pone.0216779)
Supplement: S4 Table — (PDF) [file pone.0216779.s008.pdf]

Table 4. **Means and Standard Deviations (in brackets) of the FFD, FPRT, and TRT measures, and of the proportions of RO in the complex NP region (N1+N2) and in the critical word that disambiguates the sentence (N3 region) by experimental condition and participant group.**

**Sentence region**		**N1+N2**								**N3**							
**Cognate conditions**	**Measures**	**FFD**		**FPRT**		**TRT**		**RO**		**FFD**		**FPRT**		**TRT**		**RO**	
	**Disambiguation Groups**	**HA**	**LA**	**HA**	**LA**	**HA**	**LA**	**HA**	**LA**	**HA**	**LA**	**HA**	**LA**	**HA**	**LA**	**HA**	**LA**
**C-C**	Intermediate	15.2 (4.3)	16.2 (5.2)	52.5 (13.9)	51.8 (17.5)	99.8 (51.3)	96.2 (33.8)	0.4 (0.3)	0.4 (0.3)	55.4 (13.1)	45.2 (12.4)	66.2 (20.9)	57.9 (10.1)	108.3 (53.0)	91.6 (42.7)	0.2 (0.2)	0.2 (0.2)
	Advanced	15.0 (2.9)	13.8 (3.1)	51.9 (11.1)	47.2 (13.3)	90.3 (23.5)	94.8 (43.5)	0.4 (0.2)	0.4 (0.3)	47.6 (10.9)	38.0 (7.2)	62.7 (29.7)	45.3 (11.7)	104.0 (34.1)	70.8 (23.2)	0.2 (0.2)	0.2 (0.2)
	Control	11.6 (2.3)	11.7 (2.3)	35.4 (8.8)	32.9 (8.7)	59.5 (21.0)	63.0 (22.7)	0.3 (0.2)	0.3 (0.3)	35.2 (7.5)	31.0 (5.2)	38.4 (12.4)	36.3 (6.3)	61.9 (19.7)	54.9 (18.9)	0.2 (0.3)	0.3 (0.2)
**C-NC**	Intermediate	16.1 (4.5)	16.4 (4.0)	56.5 (19.3)	58.6 (19.5)	109.0 (48.6)	101.6 (41.0)	0.4 (0.2)	0.3 (0.3)	50.1 (14.3)	57.8 (16.9)	67.8 (25.9)	69.4 (23.8)	105.2 (34.5)	116.5 (47.7)	0.1 (0.2)	0.2 (0.2)
	Advanced	12.4 (2.0)	11.8 (1.9)	50.3 (13.0)	51.4 (17.6)	98.0 (29.9)	96.2 (34.3)	0.4 (0.2)	0.5 (0.2)	50.2 (13.8)	50.6 (16.3)	58.3 (14.9)	53.0 (19.2)	99.8 (33.5)	95.1 (33.9)	0.3 (0.2)	0.2 (0.2)
	Control	12.4 (2.0)	11.8 (1.9)	36.1 (9.1)	32.5 (9.8)	63.0 (21.2)	57.4 (21.1)	0.3 (0.2)	0.3 (0.3)	36.3 (6.8)	35.0 (8.7)	40.1 (08.6)	39.5 (14.6)	65.6 (18.1)	58.1 (22.3)	0.3 (0.2)	0.2 (0.2)

**NC-C**	Intermediate	15.4 (4.7)	16.0 (4.0)	56.0 (23.3)	53.1 (15.0)	110.4 (55.8)	106.9 (62.1)	0.3 (0.3)	0.4 (0.3)	58.4 (20.2)	48.1 (13.2)	64.6 (16.1)	59.8 (16.7)	104.1 (36.6)	94.7 (39.1)	0.3 (0.2)	0.2 (0.2)
	Advanced	14.2 (2.7)	14.7 (3.4)	47.7 (15.9)	49.0 (12.7)	93.7 (37.1)	91.6 (28.6)	0.3 (0.3)	0.4 (0.2)	47.2 (9.5)	42.5 (10.7)	50.3 (10.9)	51.0 (14.3)	82.2 (26.1)	83.7 (28.9)	0.3 (0.3)	0.2 (0.2)
	Control	11.5 (1.9)	11.6 (2.3)	34.8 (9.5)	32.1 (7.5)	59.7 (18.7)	55.8 (17.8)	0.3 (0.3)	0.4 (0.3)	37.3 (6.1)	32.1 (6.2)	40.7 (7.4)	37.7 (7.1)	62.3 (24.4)	57.3 (16.4)	0.4 (0.3)	0.3 (0.2)
**NC-NC**	Intermediate	20.1 (5.8)	17.6 (7.2)	65.5 (25.1)	61.5 (19.1)	120.0 (59.2)	124.6 (80.3)	0.3 (0.3)	0.4 (0.3)	52.1 (12.2)	52.8 (14.9)	74.9 (22.7)	66.5 (13.3)	127.0 (69.8)	112.0 (46.1)	0.3 (0.2)	0.2 (0.2)
	Advanced	16.9 (4.6)	17.8 (3.6)	54.4 (18.4)	56.3 (14.6)	107.7 (40.6)	107.4 (33.0)	0.4 (0.2)	0.4 (0.2)	46.4 (10.5)	48.3 (12.4)	55.7 (12.8)	59.5 (13.8)	93.7 (28.0)	96.7 (31.8)	0.3 (0.2)	0.3 (0.1)
	Control	11.9 (1.7)	12.4 (2.1)	36.7 (10.1)	33.9 (8.9)	63.4 (23.3)	61.5 (22.9)	0.3 (0.2)	0.3 (0.3)	35.3 (06.8)	33.9 (06.5)	37.1 (05.8)	37.2 (06.1)	62.3 (16.6)	64.9 (21.1)	0.3 (0.2)	0.3 (0.2)

FFD, First Fixation Duration; FPRT, First-Pass Reading Times; TRT, Total-Reading Times; RO, Regressions Out; C-C, Cognate-Cognate; NC-C, NonCognate-Cognate; C-NC, Cognate-NonCognate; NC-NC, NonCognate-NonCognate; HA, High Attachment; LA, Low Attachment.
